# Identification and Characterization of the First Virulent Phages, Including a Novel Jumbo Virus, Infecting *Ochrobactrum* spp.

**DOI:** 10.3390/ijms21062096

**Published:** 2020-03-18

**Authors:** Przemyslaw Decewicz, Piotr Golec, Mateusz Szymczak, Monika Radlinska, Lukasz Dziewit

**Affiliations:** 1 Department of Environmental Microbiology and Biotechnology, Institute of Microbiology, Faculty of Biology, University of Warsaw, Miecznikowa 1, 02-096 Warsaw, Poland; decewicz@biol.uw.edu.pl (P.D.); m.radlinska@biol.uw.edu.pl (M.R.); 2 Department of Molecular Virology, Institute of Microbiology, Faculty of Biology, University of Warsaw, Miecznikowa 1, 02-096 Warsaw, Poland; pgolec@biol.uw.edu.pl (P.G.); mszymczak@biol.uw.edu.pl (M.S.)

**Keywords:** *Ochrobactrum* spp., virulent phage, jumbo phage, comparative genomics

## Abstract

The *Ochrobactrum* genus consists of an extensive repertoire of biotechnologically valuable bacterial strains but also opportunistic pathogens. In our previous study, a novel strain, *Ochrobactrum* sp. POC9, which enhances biogas production in wastewater treatment plants (WWTPs) was identified and thoroughly characterized. Despite an insightful analysis of that bacterium, its susceptibility to bacteriophages present in WWTPs has not been evaluated. Using raw sewage sample from WWTP and applying the enrichment method, two virulent phages, vB_OspM_OC and vB_OspP_OH, which infect the POC9 strain, were isolated. These are the first virulent phages infecting *Ochrobactrum* spp. identified so far. Both phages were subjected to thorough functional and genomic analyses, which allowed classification of the vB_OspM_OC virus as a novel jumbo phage, with a genome size of over 227 kb. This phage encodes DNA methyltransferase, which mimics the specificity of cell cycle regulated CcrM methylase, a component of the epigenetic regulatory circuits in *Alphaproteobacteria*. In this study, an analysis of the overall diversity of *Ochrobactrum*-specific (pro)phages retrieved from databases and extracted in silico from bacterial genomes was also performed. Complex genome mining allowed us to build similarity networks to compare 281 *Ochrobactrum*-specific viruses. Analyses of the obtained networks revealed a high diversity of *Ochrobactrum* phages and their dissimilarity to the viruses infecting other bacteria.

## 1. Introduction

*Ochrobactrum* spp. are non-fermenting, aerobic, Gram-negative bacteria of the *Alphaproteobacteria* class, which are frequently isolated from a variety of environmental (water, soil, and plants) and clinical (animals and human) samples [1,2,3,4,5,6,7,8]. Many strains originating from various natural and anthropogenically-shaped environments, such as wastewater or polluted soil, exhibit an extensive repertoire of enzymatic properties, which are attractive from a biotechnological perspective [9,10,11]. The reported metabolic properties include the ability to utilize many pollutants (e.g., pesticides, herbicides, and dimethylamine) and the biotransformation of toxic metal ions [12,13,14,15]. It is important to mention that some strains of *Ochrobactrum* spp. (e.g., representatives of *O. anthropii* and *O. intermedium*) are recognized as opportunistic pathogens. In these cases, bacteria are isolated mostly from immunocompromised patients, as pathogens colonizing respiratory tract and wounds. These are mostly opportunistic infections, including central venous catheter-associated bloodstream infections, prosthetic valve endocarditis, septic arthritis, osteomyelitis, and peritonitis [16,17,18,19,20]. Hence, to prevent using pathogenic or multi-resistant strains, the application of *Ochrobactrum* spp. in bioremediation or industry ought to be preceded by a careful and insightful analysis of their metabolic properties and preferentially their genomic sequences.

In 2017, the first two active lysogenic phages of *Ochrobactrum* spp. were described: a myovirus POA1180 and a podovirus POI1126 [21]. These phages were isolated together with a set of other viruses identified during an extensive screening of 125 *Ochrobactrum* isolates [21]. The performed induction experiment was successful for 60% of strains and resulted in identification of the morphologically diverse *Caudovirales* representatives. This diversity was also reflected in the host range of the induced prophages. A detailed analysis of the PAO1180 phage (originating from *O. anthropii* POA1180) showed high similarity to the prophage previously identified in the *Brucella* strain 10RB9215 isolated from an African bullfrog [22]. The annotation of the PAO1180 genome (GenBank acc. no. KX669658) revealed 58 additional genes, which are presumably relevant to its host’s metabolism (i.e., conferring resistance to chromium, mediating the uptake of sulfate or encoding predicted NAD(P) transhydrogenase). The second phage, POI1126, originating from *O. intermedium* POI1126, exhibited similarities to the *Sinorhizobium meliloti* phage PCB5, the *Erwinia pyrifoliae* phage PEp14, and the *Burkholderia cenocepacia* phages DC1, Bcep22, and BcepIL02. Interestingly, the POI1126 phage genome was nearly identical to the unnamed plasmid of the *O. intermedium* strain LMG 3301, which suggests that this phage appears in a plasmid-like form in both strains [21].

To our best knowledge, we are the first to identify two virulent phages of *Ochrobactrum* spp. For their identification, the previously characterized strain *Ochrobactrum* sp. POC9 (which efficiently enhances the utilization of sewage sludge, thereby increasing the effectiveness of biogas production) was used [11,23]. This strain was isolated from the wastewater treatment plant (WWTP) “Czajka” in Warsaw (Poland). It is a biofilm-forming and metal-resistant strain with lipolytic, proteolytic, cellulolytic, and amylolytic activities useful during the aerobic hydrolysis of complex organic compounds, such as lipids, proteins, and polysaccharides [11]. This study initially aimed to determine whether there are viruses in WWTPs that may act as natural bactericidal agents for the POC9 strain, which may limit its efficiency as a biogas production enhancer.

Both phages identified in this study, vB_OspP_OH and vB_OspM_OC, were isolated from a waste sample originating from WWTP “Wolomin” (Poland) and were subjected to further genomic and functional analyses. Moreover, a set of lysogenic phages was in silico identified in genomes of sequenced *Ochrobactrum* spp., and then a complex comparative genomic analysis of all known *Ochrobactrum* viruses was performed. This offered new insights into the overall diversity of *Ochrobactrum* phages.

## 2. Results and Discussion

### 2.1. Identification and Characterization of the Plaque and Virion Morphologies of Ochrobactrum-Specific Phages

**Figure 1:**
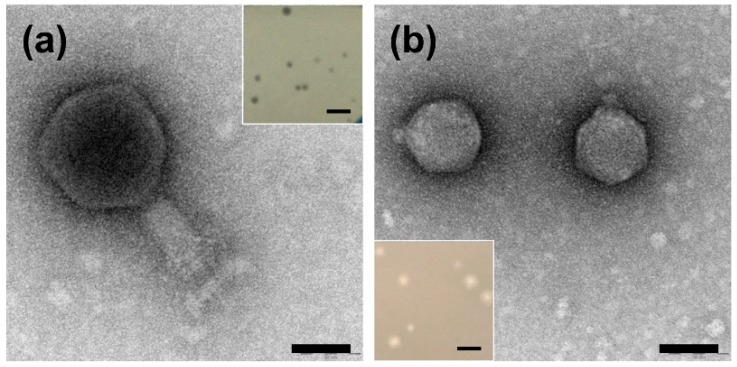
Characteristics of the virions and plaques of the vB_OspM_OC (**a**) and vB_OspP_OH (**b**) phages. The scale bars in the transmission electron microscopy (TEM) images represent 50 nm. The phage plaques are presented in the insets on panel (**a**) (plaques of vB_OspM_OC) and (**b**) (plaques of vB_OspP_OH). The scale bars in the insets represent 1 mm.

The phages that are able to infect *Ochrobactrum* sp. POC9 were isolated from a wastewater sample via the standard enrichment method [24]. After preparation of the first double layer agar plate presenting plaques, we noticed that two distinct plaque morphologies were present. Three rounds of phage propagation from a single plaque allowed us to obtain pure cultures of two different phages, initially named OC and OH, with clear plaques characterized by distinct morphologies (Figure 1). The plaques of the OC phage were smaller, albeit of irregular size in comparison to those from the OH virus. The OH phage created bigger plaques with an additional, very small (around 0.2–0.5 mm), halo zone.

Virion visualization and further morphology-based classification of the phages were carried out with the use of transmission electron microscopy (TEM). The TEM analysis of purified OC and OH phages revealed that they belong to the *Myoviridae* and *Podoviridae* families, respectively (Figure 1). Hence, according to the current naming conventions, the phages were named vB_OspM_OC and vB_OspP_OH, respectively [25]. A detailed characterization of the vB_OspM_OC and vB_OspP_OH virions’ morphologies is presented in Table 1.

### 2.2. Genomic Analysis of the vB_OspM_OC and vB_OspP_OH Phages

The vB_OspM_OC phage has a dsDNA circularly permuted genome of 227,654 bp and has a %GC content of 37.7%, which is 18% lower than the %GC content of the host POC9 genome. Such low %GC content is typical for T-even phages, particularly *Enterobacteria* T4 phages, with 35.3% GC content, while the *E. coli* (its host) %GC content is around 50% [26]. The annotation of the vB_OspM_OC genome revealed the presence of 390 putative genes and 24 tRNA genes encoding 17 different tRNAs. However, despite careful manual annotation, the predicted functions could only be assigned to 113 (29%) of the genes.

As mentioned above, the vB_OspM_OC phage carries 24 tRNA genes which cover all amino acids except of aspartic acid (Asp), phenylalanine (Phe), selenocysteine (Sec) and tyrosine (Tyr). The analysis of the vB_OspM_OC codon usage shows that, when considering all proteins being expressed, the composition of tRNAs encoded by vB_OspM_OC could cover nearly 40% of codons used during the translation. Moreover, this set of tRNAs does not seem to be correlated with low %GC content of the vB_OspM_OC genome. Interestingly, it was found, that there are only 42 (20%) or 94 (44%) of jumbo phages carrying at least 10 or only one tRNA gene, respectively. Therefore, the presence of the numerous tRNA genes is not common in jumbo phages and, as we speculate, in the case of vB_OspM_OC it may have an impact on the efficacy of its infection and propagation within the host cell.

**Figure 2:**
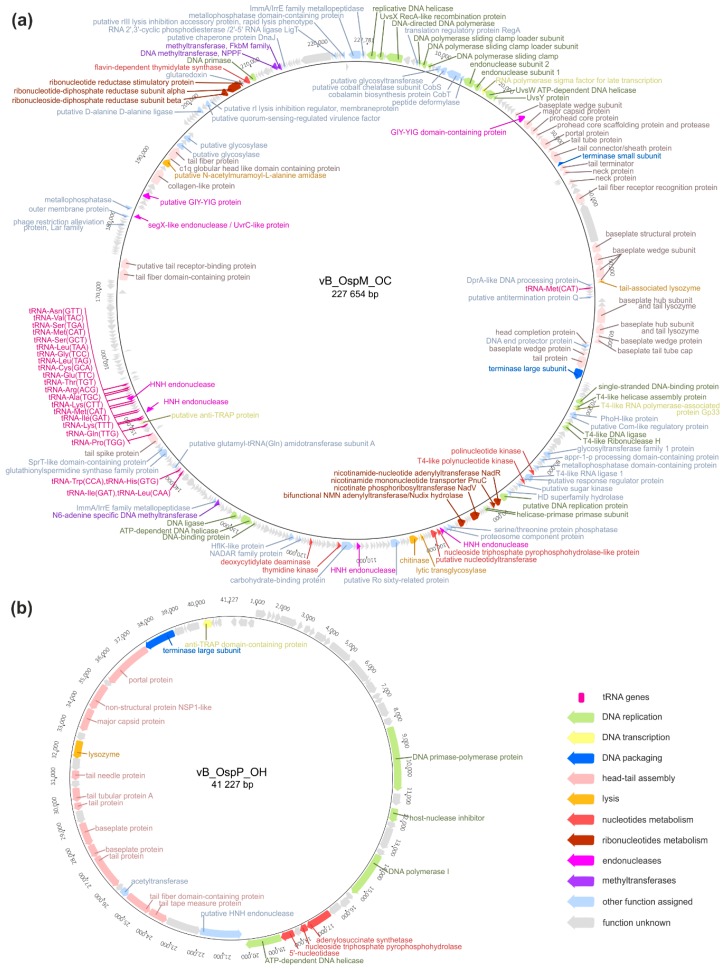
Genome organization of vB_OspM_OC (**a**) and vB_OspP_OH (**b**) phages. Arrows indicate the transcriptional orientation of the genes. All genes, except those encoding hypothetical proteins, have their predicted functions shown as labels next to their respective arrows. Additionally, the arrows were colored as presented in the legend.

The vB_OspM_OC phage has a large genome; based on the NCBI Genome database as of October 19th 2019, there were 214 phages with genomes over 200 kb, and 137 phages had genomes bigger than vB_OspM_OC. The size of a phage genome above 200 kb is an informal threshold for classifying phages as giant (so called ‘jumbo’) phages. Moreover, the phages within that group are supposed to encode the proteins responsible for DNA replication, nucleotide metabolism, several proteins for host-cell lysis, and paralogous genes encoded across different gene clusters [27,28]. Since vB_OspM_OC possesses most of these features, which are described in detail below, it can be considered a novel representative of the jumbo phages (Figure 2a).

It was also indicated that vB_OspM_OC encodes proteins that show homology with the proteins encoded by the genomes of T4-like phages. Comparative analyses revealed that vB_OspM_OC shares its core genome with this group of viruses and can thus be conceived as a novel member of the group of T4-like phages (Supplementary Table S1).

An annotation of the vB_OspM_OC genome revealed the presence of at least 21 genes encoding proteins potentially involved in replication. For 15 of them, direct counterparts of the T4 phage were identified [26,29]. Among these genes are proteins that build the replisome, such as DNA polymerase (PhiOC_p004), DNA polymerase sliding clamp (PhiOC_p010), and its subunits (PhiOC_p008-9), as well as DNA helicase (PhiOC_p001), DNA primase (PhiOC_p384), and single-stranded DNA-binding protein (PhiOC_p084). Moreover, RNase H (PhiOC_p092) and DNA ligase (PhiOC_p091) are involved in the removal of the initiator RNA primers of Okazaki fragments, and the ligation of those primers during lagging-strand DNA synthesis have also been indicated [26,30]. Potentially, vB_OspM_OC’s modes of initiating replication are similar to those of the T4 phage and are origin- and recombination-dependent [26]. This origin-dependent replication may be initiated by the predicted DNA replication protein (PhiOC_p118), which shows sequence similarity to the putative DNA replication factor of *Shewanella amazonensis* (PDB acc. no. 3BOS_B; 99.45% probability using HHPred) and the DNA polymerase III subunit beta of *Eschericia coli* O157:H7 (PDB acc. no. 5X06_G; 99.42% probability). Within this protein, AAA+-type ATPase containing the peptidase M41 domain (KOG0731; 99.35% probability) was identified based on HHpred searches. However, the exact sites involved in the priming of vB_OspM_OC DNA synthesis were not determined. The latter type of replication may be conducted by a set of DNA recombination repair proteins, such as UvsWXY homologs (PhiOC_021, PhiOC_p002, and PhiOC_022), like in the T4 phage [26,29]. The resulting DNA concatemers are required to be resolved before or during packaging, which could be conducted by endonuclease encoded by the *phiOC_p016-17* genes, which are homologs of the T4 genes, *gp46* and *gp47*.

The vB_OspM_OC also encodes at least seven genes that are potentially involved in pyrimidine nucleotide metabolism. It is possible that the predicted deoxycytidylate deaminase (PhiOH_p179; EC 3.5.4.12) converts deoxycytidylic acid (dCMP) to deoxyuridylic acid (dUMP), which can be further converted into thymidylic acid (dTMP) via flavin-dependent thymidylate synthase (PhiOC_p383; EC 2.1.1.148). Moreover, the presence of thymidine kinase (PhiOC_p167; EC 2.7.1.21) possibly helps to catalyze the conversion of (deoxy)thymidine into dTMP. Moreover, a gene encoding the nucleoside triphosphate pyrophosphohydrolase-like protein was also identified (PhiOC_p139; EC 3.6.1.9). This protein may convert nucleoside triphosphate (dNTP) into a nucleotide (dNMP) and diphosphate (PP_i_). Moreover, genes encoding two polynucleotide kinases (PhiOC_p107 and PhiOC_p110) and a putative nucleotidyltransferase (PhiOC_p140) were also identified, which together take part in the nucleotide salvage pathway [30,31].

Another feature typical for jumbo phages is nicotinamide adenine dinucleotide (NAD^+^) metabolism. The vB_OspM_OC phage encodes at least seven proteins that are potentially involved in that process. The presence of bifunctional NMN adenylyltransferase (NAmPRTase)/Nudix hydrolase (NatV; PhiOC_p132; EC 2.4.2.12/EC 3.6.1.22) and nicotinate phosphoribosyltransferase (NadV; PhiOC_p128; EC 6.3.4.21) possibly allows the vB_OspM_OC phage to convert nicotamide to nicotamide D-ribonucleotide and nicotinate into nicotinate D-ribonucleotide, respectively. Both of these ribonucleotides can be further transformed into NAD^+^ or deamino-NAD^+^, respectively, via nicotinamide–nicleotide adenylyltransferase (NadR; PhiOC_p119; EC 2.7.7.1). The Nudix hydrolase domain of NatV may also help reverse the last reaction and transform deamino-NAD^+^ into a nicotamide D-ribonucleotide. The presence and activity of NatV and NatD encoded by the *Vibrio* phage KVP40 were recently shown to be sufficient for the NAD^+^ salvage pathway [32]. Moreover, it was shown that at least 169 different bacteriophages encode the NadV-like NAmPRTase. However, only 50 documented phages encode a bifunctional NatV-like enzyme, and all of these also contain a *nadV* gene in their genomes [30]. Amongst them, 21 phages have genomes with sizes above 200 kb, including the previously mentioned KVP40 phage. Therefore, the vB_OspM_OC phage could be another example of a giant phage extending the group of viruses with both NatV and NatD. However, their activity requires experimental validation during future studies. The vB_OspM_OC phage also encodes the predicted nicotinatinamide mononucleotide transporter PnuC (PhiOC_p120), which is involved in the NAD^+^ biosynthesis pathway. Furthermore, three other genes encode ribonucleotide-diphosphate reductase (rNDP; PhiOC_p379 and PhiOC_p380; EC 1.17.4.1) and ribonucleotide reductase (RNR; PhiOC_p381; EC 1.17.4.2), which possibly catalyze the formation of deoxyribonucleotides from ribonucleotides and can further be used in the synthesis of DNA [31].

The vB_OspM_OC virion is relatively large, and 33 out of 113 proteins with recognized functions are possibly involved in its formation. Eleven of these proteins were identified as baseplate and/or wedge proteins that may correspond to the firm and complex structure of the baseplate shown on the micrographs (Figure 1a). Within four of these structural proteins, the domains involved in host cell lysis were also identified, including the tail-associated lysozymes in PhiOC_p064 and PhiOC_p065, the glycoside hydrolase domain in PhiOC_p326 (annotated as a putative tail receptor-binding protein), and endo-*N*-acetylneuraminidase in a tail fiber protein (PhiOC_p358). The repertoire of lysing enzymes of vB_OspM_OC is extended by the putative *N*-acetylmuramoyl-L-alanine amidase (PhiOC_p356), lytic transglycosylase (PhiOC_p145), and chitinase (PhiOC_p147), of which the latter two were not located within structural gene clusters.

Interestingly, within the vB_OspM_OC genome, a predicted N6-adenine (m6A) DNA methyltransferase gene (PhiOC_p213) was found. As shown previously, viruses infecting *Alphaproteobacteria* often have DNA methyltransferases (MTases) that exhibit GANTC (methylated nucleotide is underlined) specificity, just as the host-encoded cell cycle regulated MTase CcrM [33,34,35]. To determine whether GANTC sequences are substrates for PhiOC_p213, we digested the pET_PhiOC_p213 plasmid DNA isolated from IPTG-induced and non-induced *E. coli* cultures with the HinfI restriction enzyme (specificity GANTC, inhibited by m6A methylation). The DNA isolated from the induced cultures was resistant to cleavage by HinfI but sensitive to cleavage with the various enzymes (e.g., Csp6I and Hin1II) used as controls. The pET_PhiOC_p213 DNA isolated from the non-induced cultures was susceptible to all restriction enzymes used, including HinfI.

DNA MTases, which are related to PhiOC_p213, are encoded within several other phages, including the *Silicibacter* phage DSS3-P1 (GenBank acc. no. AET42337.1) and the *Loktanella* phage pCB2051-A (GenBank acc. no. AGH31487.1), with a ~45% protein sequence identity. It is worth mentioning that, in contrast to previously characterized phage-encoded MTases with GANTC specificity (such as PhiLM21_p027 [35], phi2LM21_p21, and phi3LM21_p21 [33], which do not share sequence similarities with the host-encoded CcrM enzymes), PhiOC_p213 exhibits 30.49% identity with CcrM of *Ochrobactrum* sp. POC9 (GenBank acc. no. PWU77214.1) and shows 29% identity with for example CcrM of *O. pseudogrignonense* K8 (GenBank acc. no. ANG95423.1), for which methylation specificity was shown by a PacBio analysis (as found in REBASE: http://rebase.neb.com/cgi-bin/pacbioget?19606). This sequence conservation is limited to the N-terminal methyltransferase domain of CcrM, since a C-terminal nonspecific DNA-binding domain (~80 residue segment) is present only in bacterial CcrM MTases (Supplementary Figure S1).

The co-occurrence of phage- and host-encoded enzymes with the same sequence specificity mimicking the host strategies in DNA methylation by viruses is a known phenomenon discovered not only in *Alphaproteobacteria* [33] but also in *Gammaproteobacteria*. The dam-like activity (the methylation of GATC to Gm^6^ATC in *Gammaproteobacteria*) of phage MTases has been shown for, e.g., P1 [36], VT-2 [37] and the T-even coliphages [38], but only DamT2 and DamT4 are homologs of the cellular (*E. coli* in this case) Dam enzyme [39], like the above mentioned PhiOC_p213 and CcrM of *Ochrobactrum* sp. POC9. The potential functions of the maintenance of both, the specificity of the T4-like phage and the bacterial CcrM (in *Alphaproteobacteria*) and Dam MTases (in *Gammaproteobacteria*), as well as the conservation of the amino acid sequences of these proteins in virus–host biology, remain to be elucidated.

The second isolated phage, vB_OspP_OH, has a dsDNA genome, with a size of 41,227 bp. In contrast to vB_OspM_OC, its %GC content is 55.2%, which is nearly the same as the content of the host strain. Within its genome, 65 potential protein encoding genes were identified, of which 23 (35.4%) had their functions predicted. The vB_OspP_OH phage seems to be unique, showing only limited similarities with the viruses recovered from the environmental samples collected by Mizuno and coworkers, (i.e., the uncultured Mediterranean phage uvMED [40]).

Amongst the predicted proteins of vB_OspP_OH, there are three involved in phage genome replication, including a potential bifunctional DNA primase-polymerase protein (PhiOH_p22), DNA polymerase I (PhiOH_p28), and DNA helicase (PhiOH_p36) (Figure 2b). The sensitive searches with HHpred allowed identification within the PhiOH_p22 protein the primase-polymerase domain (cd04859; 99.4% confidence) in its N terminal part (positions 39–217) and the phage- or plasmid-associated DNA primase domain (100% confidence) on its C terminus (positions 452–813).

Within the genome of vB_OspP_OH, three genes encoding proteins potentially involved in nucleotide metabolism were found (Figure 2b). These proteins are adenylosuccinate synthase (PhiOH_p32, EC 6.3.4.4), adenosine triphosphate pyrophosphatase (PhiOH_p33, EC 3.6.1.8), and 5′-nucleotidase (PhiOH_p35, EC 3.1.3.5). Their presence potentially allows vB_OspP_OH to transform nucleotides into nucleosides, resulting in the salvage of (pyro)phosphate groups for further reuse in dNTP synthesis during phage genome replication [31].

The virion structure of vB_OspP_OH allows its classification as a podovirus. Within the vB_OspP_OH genome, 10 genes encoding structural proteins were identified (Figure 2b). These include: (i) a portal protein (PhiOH_p57), (ii) a scaffolding protein (PhiOH_p55), (iii) a major capsid protein (PhiOH_p54), (iv) a tail tubular protein A (phiOH_p48), (v) a minor tail protein (phiOH_p47), (vi) a tail fiber protein (PhiOH_p45), (vii) a tail protein (PhiOH_p43), (viii) a tail fiber protein (PhiOH_p42), (ix) a tail-tape measure protein (PhiOH_p42), and (x) a tail tape measure protein (PhiOH_p39). Within the last protein, the peptidoglycan hydrolase (EtgA) domain was identified using HHpred. This protein could be responsible for the presence of the plaque halo effect observed for this phage (Figure 1b).

The assumed endolysin of vB_OspP_OH (PhiOH_p52) shows the highest similarity to the *Ochrobactrum* phage POI1126 secretion activator protein (APU92960.1, 43.3% sequence identity). An analysis of the domains of PhiOH_p52 and APU92960.1 revealed the presence of the glycosyl hydrolase 108 domain (pfam05838, e-value below 1e-30), which acts as a lysozyme [41].

### 2.3. Comparative Genomics of Ochrobactrum Phages

**Figure 3:**
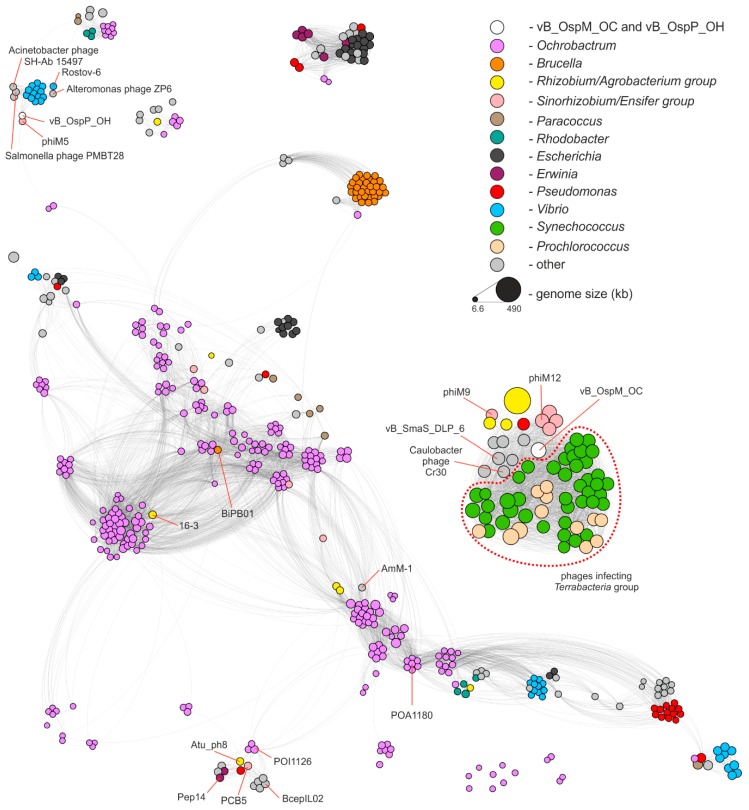
Protein-based similarity network of *Ochrobactrum* (pro)phages to the other selected phages. Within the network, constructed with vConTACT v2 and visualized in Gephi, each node (575 in total) represents a (pro)phage, and each edge (9622) corresponds to the vConTACT’s degree of similarity between the two phages. The size of the nodes reflects the size of the (pro)phage genome, and its color corresponds to the host genus (if at least five viruses infected by the same genus were included in the network). Nodes representing the vB_OspM_OC and vB_OspP_OH phages are presented in white. If less than five phages were included in the network, they were presented in a joint category called “other”; these were phages infecting *Ralstonia*, *Burkholderia*, *Stenotrophomonas*, *Wolbachia*, *Achromobacter*, *Sulfitobacter*, *Pasteurella*, *Enterobacter*, *Klebsiella*, *Shigella*, *Roseobacter*, *Salmonella*, *Rhodovulum*, *Thiobacimonas*, *Xantomonas*, *Pelagibacter*, *Dinoroseobacter*, *Mesorhizobium*, *Cystobacterineae*, *Alteromonas*, *Acidithiobacillus*, *Azospirillum*, *Alteromonadaceae*, *Serratia*, *Delftia*, *Acinetobacter*, *Aurantimonas*, *Enterobacteria*, *Caulobacter*, *Acidovorax*, and *Bradyrhizobium*. Additionally, the phages infecting the *Terrabacteria* group were distinguished with a dotted line.

Both phages characterized in this study are distinct from the viruses identified so far, with a protein sequence identity not exceeding 70% for vB_OspM_OC (PhiOC_p379) or 59% for vB_OspP_OH (PhiOH_p53), when at least a 75% sequence coverage was considered. To perform a complex analysis of the diversity of *Ochrobactrum* phages, the PhiSpy v3.4 tool (trained with the complete *Ochrobactrum* spp. genomes and a draft genome of *Ochrobactrum* sp. POC9, in which the prophage regions were indicated manually) was applied. This enabled us to significantly extend the knowledge in this field since, in the previous works by Jackel et al. [21,42,43] a total of 66 *Ochrobactrum* (pro)phages were indicated, while in our analysis, 277 prophages were identified within 104 out of the 113 analyzed genomes (Supplementary Table S2). Moreover, by applying the vConTACT v2 tool [44], we compared vB_OspM_OC, vB_OspP_OH, and all identified *Ochrobactrum* prophages with the ProcaryoticViralRefSeq85-Merged database resources and the 12,817 bacteriophages present in the NCBI Genome database (as of 10 July 2019). This resulted in the identification of 294 phages similar to *Ochrobactrum* spp. (pro)phages. A comparison of all those (pro)phages is presented in the form of a similarity network composed of 575 (pro)phages in total (Figure 3).

The distribution of (pro)phages (represented by nodes) in the network allows for more precise insight into *Ochrobactrum* (pro)phages diversity. It was shown that 198 (70.5%) *Ochrobactrum* (pro)phages shared similarity to the phages infecting other *Proteobacteria* (223 (pro)phages in the network), which is representatives of the *Terrabacteria* group (54) or of phages recovered from the metagenomic samples for which hosts were not assigned (17). Amongst the *Proteobacteria* (pro)phages grouped together with *Ochrobactrum* phages, 89 viruses infected *Alphaproteobacteria*, 15 *Betaproteobacteria*, and 115 *Gammaproteobacteria*. As for the *Terrabacteria* group, these phages were recognized as infecting agents only for cyanobacteria, representing the *Synechoccoccus* (42 viruses) and *Prochlorococcus* (12) genera. It was also shown that 75 (26.7%) *Ochrobactrum* viruses shared homologous proteins exclusively with other *Ochrobactrum* prophages, and 8 viruses formed orphan nodes, which exemplifies the uniqueness of these (pro)phages.

This global analysis also allowed us to determine the relatives of the vB_OspM_OC and vB_OspP_OH phages. vB_OspM_OC is related to large, mostly T4-like, phages. Its highest similarity was observed to be with myoviruses (i.e., the *Sinorhizobium* phages phiN3, phiM7, phiM12, and phiM19), *Stenotrophomonas* phage vB_SmaS_DLP_6, and *Caulobacter* phage Cr30 (GenBank acc. nos.: NC_028945.1, NC_041929.1, NC_027204.1, KR052481.1, KU682439.2, and NC_025422.1, respectively) [45,46]. Interestingly, vB_OspM_OC seems to also be related to the phages infecting the *Terrabacteria* group of bacteria. This may suggest their common origin.

vB_OspP_OH created a separate cluster with another podovirus, the genomically highly mosaic *Sinorhizobium meliloti* phage phiM5 [45]. Direct comparison of these phages (Supplementary Figure S2) indicated that both viruses encode homologous structural proteins. However, other functionally equivalent proteins, like bifunctional DNA primase/polymerase, were also found. Interestingly, vB_OspP_OH also showed similarities to the group of *Vibrio* podoviruses (e.g., phiVC8, Rostov-6) and the *Alteromonas* phage ZP6, as well as two siphoviruses the *Salmonella* phage PMBT28 and the *Acinetobacter* phage SH-Ab 15497 (GenBank acc. nos.: NC_027118.1, MH105773.1, MK203850.1, MG641885.1, and MG674163.1, respectively) [47,48].

### 2.4. Functional Characterization of vB_OspM_OC and vB_OspP_OH

#### 2.4.1. Host Range Analysis

Various bacterial strains (29 in total), including the reference ones (*Agrobacterium tumefaciens* LBA288, *Escherichia coli* C600, *E. coli* DH5α, *E. coli* K-12, *E. coli* BR825, *Paracoccus alcaliphilus* JCM 7364, *Paracoccus aminophilus* JCM 7686, *Pseudomonas aeruginosa* PAO1161, and *Variovorax paradoxus* EPS), the isolates from wastewater treatment plants (*Bacillus* sp. LPOC3, *Bacillus* sp. LPSUB4, *Brevundimonas* sp. POC21, *Klebsiella* sp. POC16, *Lysinibacillus* sp. LPSUB15, *Rummelibacillus* sp. POC4, and *Stenotrophomonas* sp. POC10), and other environmental isolates (*Achromobacter* sp. LM16R, *Brevundimonas* sp. LM18, *Brevundimonas* sp. LPMIX5, *Ensifer* sp. M14, *Janthinobacterium* sp. M1_6, *Janthinobacterium* sp. M1_18, *Janthinobacterium* sp. M2_12, *Janthinobacterium* sp. W1_1, *Ochrobactrum* sp. LM19, *Pseudomonas* sp. LM7, *Psychrobacter* sp. DAB_AL32B, *Sinorhizobium* sp. LM21, and *Sphingomonas* sp. WLOD2_3), were used in the host range assay analysis. The host range and lytic activity of the vB_OspM_OC and vB_OspP_OH phages were assessed using spot test assays. Even though various bacteria representing the 18 genera were used in the assay, we observed clear zones only on the plates with *Ochrobactrum* sp. POC9 and *Ochrobactrum* sp. LM19. The comparison of plaques obtained for LM19 and POC9 showed no differences in the number of plaques (when performing a spot test assay using a series of various concentrations of phages) and in their morphology. Both analyzed phages produced plaques on both *Ochrobactrum* species. This suggests that both phages are specific to *Ochrobactrum* spp.

#### 2.4.2. Adsorption Assay and One-Step Growth Curve

**Figure 4:**
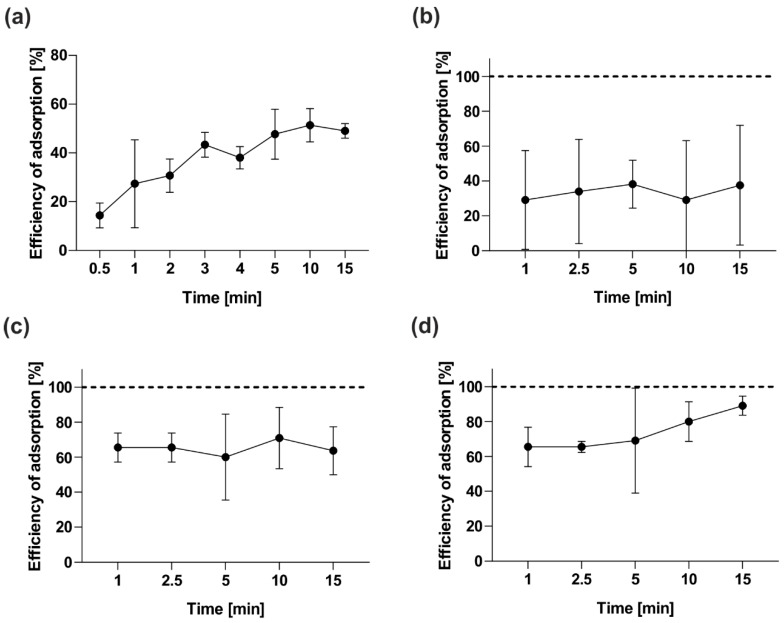
Adsorption efficiency of the vB_OspM_OC and vB_OspP_OH phages. The experiment with an overnight culture of *Ochrobactrum* sp. POC9 infected by vB_OspP_OH and vB_OspM_OC is shown on panels (**a**) and (**b**), respectively. In the case of vB_OspM_OC, tests were also performed using the POC9 cultures with OD_600_ = 0.2 (**c**) and OD_600_ = 0.4 (**d**). The presented results are the average values from three experiments, with standard deviation represented by the error bars.

The efficiency of phage development depends on two main steps: the phage’s adsorption to the bacteria and its development inside the host cell. Adsorption assays of vB_OspM_OC and vB_OspP_OH were performed to analyze the kinetics of their adsorption to *Ochrobactrum* sp. POC9. First, we carried out an analysis with the use of bacteria after overnight culturing. The results of that experiment (Figure 4a,b) revealed that 25% of both phages attached to the host cells within 1 min. However, after 5 min, an increase of the adsorbed phages to around 50% was observed only in the case of vB_OspP_OH. The attachment efficiency of vB_OspM_OC was stable, and the amount was around 25% during the whole experiment. Since vB_OspM_OC adsorbed inefficiently to the bacteria from the overnight culture, the adsorption assay for this phage was repeated using actively growing bacteria (Figure 4c,d). We observed that 65% of the vB_OspM_OC attached to the host cells within 1 min in both experimental variants (with initial OD_600_ = 0.2 and OD_600_ = 0.4). Moreover, around 90% of the vB_OspM_OC phages were adsorbed into the host cells in 15 min, but only when a bacterial culture of OD_600_ = 0.4 was used. Otherwise, the attachment efficiency of vB_OspM_OC was stable, and the amount was around 65% during the whole experiment.

**Figure 5:**
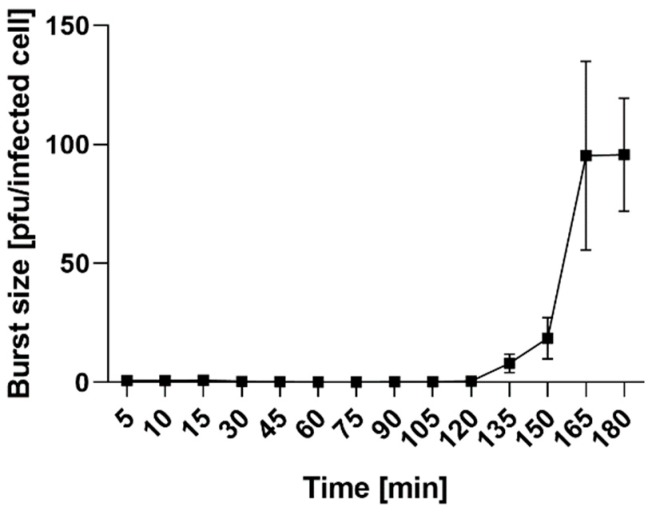
One-step growth curve of the vB_OspP_OH phage. The presented results are the average values from three experiments, with the standard deviation represented by error bars.

The phage growth parameters were determined with the use of a one-step growth curve assay. The latent period of vB_OspP_OH is 135 ± 5 min, and the burst size of one lytic cycle is approximately 95 ± 10 pfu per infected cell (Figure 5). The vB_OspM_OC phage was more difficult to analyze, and, despite several repeats of the one-step growth experiment, we were unable to obtain conclusive and replicable results (Supplementary Figure S3). Therefore, the latent period and burst size were not determined.

We speculate, that this ‘problematic’ propagation of the vB_OspM_OC phage could be explained by its complex development strategies. As determined previously, the vB_OspM_OC belongs to the T4-like group of viruses, and its genome may contain genes encoding proteins that enable diverse modes of phage development, such as the T4 phage. It was shown that T4 can develop by applying alternative mechanisms, such as lysis inhibition (LIN) [49] or pseudolysogeny [50]. It is possible that various (possibly not yet described) development strategies may also be characteristic for vB_OspM_OC, especially since the functions of most proteins that it encodes remain still unknown. An alternative, speculative explanation could be the activity of a phage infection defensive mechanism in *Ochrobactrum* sp. POC9. The manual annotation of the POC9 prophage regions identified an abortive infection protein (GenBank acc. no. WP_109988358.1) encoded within vB_OspX_pp134 (Supplementary Table S2). This kind of defensive mechanism allows successful phage entry but interrupts one of the essential processes in the phage lifecycle, which prevents the release of functional virions. This induces infected bacterial cell death and protects the bacterial population from further infections [51,52].

#### 2.4.3. Killing Assay

To assess the killing activity of vB_OspM_OC and vB_OspP_OH on *Ochrobactrum* sp. POC9, bacterial growth was monitored by measuring the optical densities of OD_600_ after phage infection. To evaluate the efficiency of phage development, various concentrations of phages were used for infection (from 10^2^ to 10^10^ pfu/mL, which corresponds to MOI approximately from 0.0000001 to 10) (Supplementary Figure S4).

Both phages were able to cause lysis of the liquid bacterial culture, especially when the phage lysates with the highest concentrations (10^8^–10^10^ pfu/mL) were used (Supplementary Figure S4). In these cases, the optical density of the cultures decreased at approximately 100–400 min post-infection. When lower concentrations of phages were used (≤10^7^ pfu/mL), a decrease in optical density was observed (except of 10^2^ and 10^3^ pfu/mL for vB_OspM_OC), albeit at a longer time after infection (Supplementary Figure S4).

#### 2.4.4. Stability of the vB_OspM_OC and vB_OspP_OH Phages

**Figure 6:**
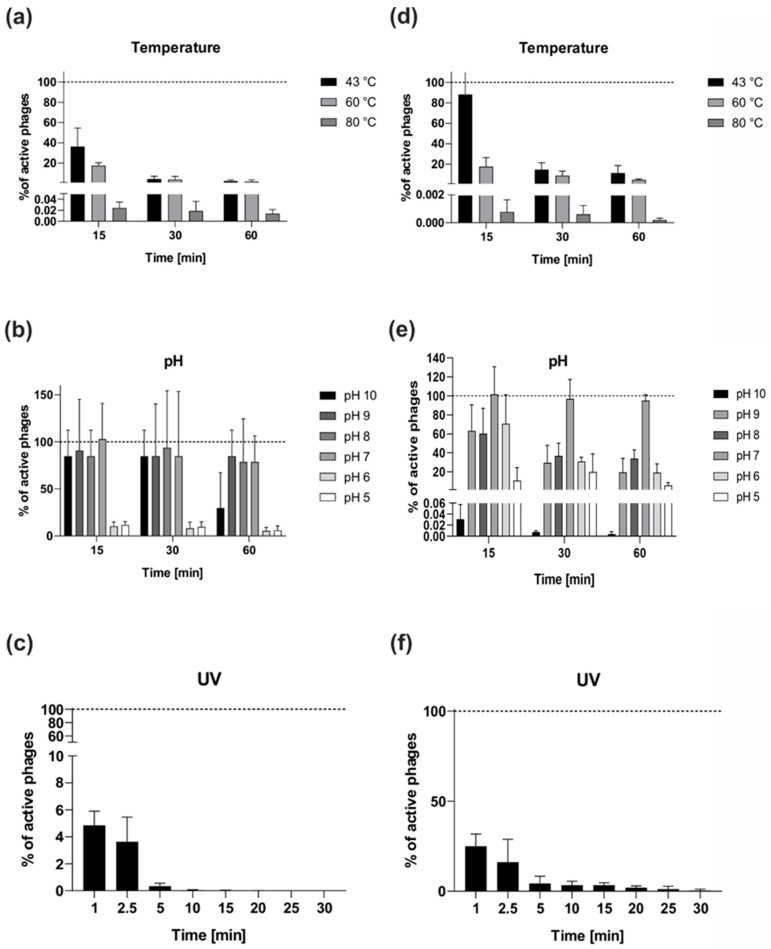
Stability of the vB_OspM_OC (left column) and vB_OspP_OH (right column) phages at various temperatures (**a** and **d**, respectively), pH (**b** and **e**, respectively)**,** and UV light (**c** and **f**, respectively). The presented results are the average values from three experiments, with standard deviation represented by the error bars.

Storage, transfer, and downstream experiments with the phages require information about their stability. vB_OspM_OC seems to be temperature-sensitive and exhibited a decrease of its phage titer to approximately 50% after incubation at 43 °C for 15 min (Figure 6a). After incubation for an hour at all chosen temperatures, there was almost a complete reduction in the vB_OspM_OC titer (Figure 6a). In contrast, vB_OspP_OH seems to be more temperature-resistant (Figure 6d). It was observed that incubation of vB_OspP_OH at 43 °C for 15 min does not influence the phage titer. However, a longer incubation at 43 °C results in the inactivation of approximately 85% of phages. Only around 18% of vB_OspP_OH particles were able to survive at 60 °C (Figure 6d).

Both phages appeared to be stable at pH values from 7 to 8 (Figure 6b,e). However, vB_OspM_OC showed higher stability at a higher pH (pH from 9 to 10) (Figure 6b), whereas vB_OspP_OH showed higher stability at lower pH values (pH from 5 to 6) (Figure 6e).

An analysis of phage particle stability after UV light treatment showed that both phages are UV-sensitive (Figure 6c,f). However, vB_OspP_OH showed higher resistance in comparison to vB_OspM_OC (Figure 6f). The plaques of vB_OspP_OH was observed even after 30 min of exposure, while vB_OspM_OC was completely inactivated (no plaques were observed) after 20 min of UV exposition (Figure 6c).

## 3. Materials and Methods

### 3.1. Bacterial Strains, Plasmids and Culture Conditions

The following bacterial strains were used: *Achromobacter* sp. LM16R [53], *Agrobacterium tumefaciens* LBA288 [54], *Bacillus* sp. LPOC3 (laboratory collection), *Bacillus* sp. LPSUB4 (laboratory collection), *Brevundimonas* sp. LM18 [53], *Brevundimonas* sp. LPMIX5 [23], *Brevundimonas* sp. POC21 (laboratory collection), *Ensifer* sp. M14 [55], *E. coli* C600 (laboratory collection), *E. coli* DH5α [56], *E. coli* K-12 (laboratory collection), *Janthinobacterium* sp. M1_6 (laboratory collection), *Janthinobacterium* sp. M1_18 (laboratory collection), *Janthinobacterium* sp. M2_12 (laboratory collection), *Janthinobacterium* sp. W1_1 (laboratory collection), *Klebsiella* sp. POC16 (laboratory collection), *Lysinibacillus* sp. LPSUB15 (laboratory collection), *Ochrobactrum* sp. LM19 [53], *Ochrobactrum* sp. POC9 [11], *Paracoccus alcaliphilus* JCM 7364 [57], *Paracoccus aminophilus* JCM 7686 [58], *Pseudomonas aeruginosa* PAO1161 [59], *Pseudomonas* sp. LM7 [60], *Psychrobacter* sp. DAB_AL32B [61], *Rummelibacillus* sp. POC4 [23], *Sinorhizobium* sp. LM21 [53], *Sphingomonas* sp. WLOD2_3 (laboratory collection), *Stenotrophomonas* sp. POC10 (laboratory collection), and *Variovorax paradoxus* EPS [62]. These strains were grown on the LB medium at 37 °C (*E. coli*) and 30 °C (all other strains). The medium was solidified by the addition of 1.5% (*w*/*v*) agar. Where necessary, the medium was supplemented with X-gal, IPTG, and kanamycin (50 μg/mL). Plasmid pET30a was used for cloning of the DNA methyltransferase gene.

### 3.2. DNA Manipulations and Introduction of Plasmid DNA into Bacterial Cells

Standard DNA manipulations were performed as described by Sambrook and Russell (2001) [63]. Plasmids constructed in this study were introduced into *E. coli* ER2566 by chemical transformation [64].

### 3.3. Cloning, Overexpression, Purification, and Testing of Putative DNA MTase Activities

The predicted DNA MTase gene identified within the vB_OspM_OC phage was amplified by PCR using a Thermo Scientific™ Phusion™ High-Fidelity DNA Polymerase (Thermo Fisher Scientific, Inc., Waltham, MA, USA) and following the primer pairs OcFnde (5′-GTTGTTCATATGGAAAATTTGACACTTTTTAATGGTAAC-3′) and OcRxho (5′-GTTGTTAATAAAAATAGGCGTTAGCTCACC-3′). DNA amplification was performed using a Mastercycler (Eppendorf, Hamburg, Germany). Each thermocycle started with an initial denaturation at 95 °C for 1 min followed by 30 cycles of denaturation at 98 °C for 10 s, annealing at 66 °C for 20 s, extension at 72 °C for 30 s, and finished with a final extension at 72 °C for 2 min. The PCR product (after purification) was digested with NdeI and XhoI and ligated with a pET30a vector cut with the same enzymes. The recombinant proteins were expressed in the *E. coli* ER2566. The protein expression and restriction enzyme digestion protection assays for revealing the sequence specificity of the tested MTase were performed as previously described [33,65].

### 3.4. Bacteriophages Isolation

Both phages, vB_OspP_OH and vB_OspM_OC, were isolated from a wastewater sample collected from a sewage treatment plant in Wołomin (Wołomin, Masovian Voivodship, Poland) on 25 August 2018. Phage isolation was performed according to the previously described procedure [24]. After the isolation, three rounds of propagation from each individual phage plaque were carried out to prepare a lysate of the pure phage strain.

### 3.5. Transmission Electron Microscopy (TEM)

Transmission electron microscopic images of the phage virions were obtained using a TEM LIBRA 120 (Zeiss, Oberkochen, Germany) microscope (HT = 120 kV, LaB6 cathode). Samples were prepared by applying a 10 μL droplet of phage suspension to thin, carbon-coated copper grids (400 mesh), followed by immersing the grids in 1% uranyl acetate for contrasting. Then, the samples were left to dry at room temperature. The visualization of the phages was performed at the Laboratory of Theory and Applications of Electrodes, Faculty of Chemistry, University of Warsaw, Poland.

### 3.6. Host Range Analysis

To determine the host range of the vB_OspM_OC and vB_OspP_OH phages, 2.5 μL of each phage lysate was spotted on double layer agar plates with various tested bacteria in the upper layer. The appearance of the plaques was observed after an overnight incubation of the plates at 30 °C.

### 3.7. Adsorption Kinetics Assay of vB_OspM_OC and vB_OspP_OH

The adsorption assay was conducted as described previously [66]. Briefly, 1 mL of an overnight or logarithmic *Ochrobactrum* sp. POC9 culture was spiked with 10 µL of the phage suspension (around 10^9^ pfu/mL). The mixture was incubated without shaking at 37 °C. After 1, 2.5, 5, 10, and 15 min, 100 µL aliquots were withdrawn and centrifuged (5000× *g*, 1 min, RT) to deposit the phage-adsorbed cells as sediment. The titer of the remaining free phages was determined by supernatant titration on double-layer agar plates. The initial number of phages (100% of the phages used) was determined by adding an appropriate volume of the vB_OspM_OC or vB_OspP_OH phage lysate to a medium without bacteria, followed by titration. The number of adsorbed phages was determined via the decrease in PFUs in the supernatant relative to the initial number of phages. The data were obtained from three independent experiments.

### 3.8. Killing Assay

For the host culture collapse studies, liquid *Ochrobactrum* sp. POC9 cultures were infected with vB_OspM_OC or vB_OspP_OH phage lysates. Briefly, an overnight culture of bacteria was refreshed in an LB medium and allowed to grow until reaching an OD_600_ of 0.25. Then, the culture was infected with the vB_OspM_OC or vB_OspP_OH phage lysate at various concentrations ranging from 10^2^ to 10^10^ pfu/mL. The OD_600_ measurement was made every 15 min for 24 h. Measurements were carried out with the use of an Infinite 200 PRO multimode plate reader and microplate reader software v 1.8 2010 (Tecan, Männedorf, Switzerland). The data were obtained from three independent experiments.

### 3.9. One-Step Growth Curve

For a one-step growth analysis, 25 mL of LB medium was inoculated with an overnight culture of *Ochrobactrum* sp. POC9 (50:1, *v*/*v*). The culture was grown at 37 °C with shaking (150 rpm) until OD_600_ = 0.2 and then infected with the vB_OspP_OH phage at MOI of 0.1 and incubated without shaking at 37 °C for 5 min. Next, 1 mL of the infected bacteria was centrifuged at 5000 rpm for 1 min at 37 °C. The resulting pellet was washed and re-suspended in 1 mL of fresh LB broth. Then, 25 µL of infected cells was transferred to a fresh LB broth and incubated at 37 °C with shaking (150 rpm). At appropriate time points, the PFUs (300 µL of samples treated with 300 µL of chloroform) were calculated. The samples for estimation of the number of infection centers (ICs) and the burst size presented as pfu/IC were analyzed as described previously [49]. The data were obtained from three independent experiments.

### 3.10. Bacteriophage Stability at Various Values of pH and Temperature

For pH stability testing, 10 µL of the vB_OspM_OC or vB_OspP_OH phage lysate (1 × 10^9^ pfu/mL) was mixed with 990 µL LB broth in a series of tubes, each with a different pH (adjusted using NaOH or HCl), and incubated for 10, 30, or 60 min at room temperature. For temperature stability testing, 10 µL of the phage lysate (1 × 10^9^ pfu/mL) was mixed with 990 µL LB broth in a series of tubes, and the samples were incubated at 43, 60, or 80 °C for 15, 30, or 60 min. At each of these time-points, the number of phages (plaque forming units, PFUs) was calculated. The control phage samples were incubated at pH 7 and room temperature (22 °C), respectively. The data were obtained from three independent experiments.

### 3.11. Bacteriophage Resistance to UV Light

For UV resistance testing, 10 µL of the vB_OspM_OC or vB_OspP_OH phage lysate (1 × 10^9^ pfu/mL) was mixed with 990 µL of LB broth. The mixture was then spotted onto a polystyrene Petri dish and exposed to UV-C light (UVC lamp, Philips TUV-30-W-245 nm Lamp, type No. 57413-P/40) from a distance of 50 cm for 1, 2.5, 5, 10, 15, 25, and 30 min. A control sample was incubated on a laboratory bench at room temperature. The data were obtained from three independent experiments.

### 3.12. DNA Sequencing

DNA sequencing was performed using an Illumina MiSeq instrument (Illumina, San Diego, CA, USA) in the paired-end mode using a v3 chemistry kit (Illumina). The obtained sequence reads were filtered for quality using fastp v0.19.5 with a window size of 10 bps moving from 5′ to 3′ and removing bps with a quality lower than 15, polyX at the 3′ end, and the reads of lengths lower than 150 bps [67]. Afterwards, the reads were assembled using SPAdes v3.11.1 with default parameters [68]. The presence of redundant ends in the assembled contigs was verified by remapping the reads against the assembled genomes using bwa mem v0.7.17-r1188, processed afterwards with samtools v1.7 and viewed in the Integrative Genome Viewer v2.6.2 [69,70,71]. Circulation of the genomes was validated by capillary sequencing of the PCR products using an ABI3730xl DNA Analyser (Applied Biosystems, Waltham, MA, USA).

### 3.13. Detection of Prophages in Bacterial Genomes and Classification of Phages

On October 19th 2019, 125 genomes of *Ochrobactrum* spp. were retrieved from the National Center for Biotechnology Information (NCBI) genome browser (Supplementary Table S2). Twelve genomes were removed from the analysis due to the lack of annotations. From among the remaining 113, six complete genomes (*O. anthropi* strains ATTC 49188, OAB and T16R-87, *O. pseudogrigronense* K8, *O. pituitosum* AA2, and *O. quorumnocens* A44) and a draft genome of *Ochrobactrum* sp. POC9 were manually inspected for the presence of the prophage regions (Supplementary Table S2). These were indicated and provided as additional training sets for PhiSpy v3.4 [42,43]. Then, retrained PhiSpy was used for the screening of prophage regions within the remaining 106 draft genomes of *Ochrobactrum* spp. The prophage was distinguished when at least 20 phage-like genes were found in the particular DNA region. Identification of the prophages was additionally validated by searching the pVOG database HMM profiles against the extracted regions using hmmsearch v3.1b2 [72] and applying cut-offs for sequence e-value—1e-10 and domain e-value—1e-5, followed by manual inspection [72,73].

The taxonomic assignment of all (pro)phages was conducted based on the results of vConTACT v2 with the use of the ProcaryoticViralRefSeq85-Merged database applying DIAMOND v0.9.24 for protein clustering and ClusterOne v1.0 for protein and viral cluster analyses [44,74,75].

### 3.14. Genome Annotation

The manual annotation of the analyzed phage genomes was conducted using Artemis software [76]. First, possible genes were indicated using PHANOTATE [77]. The annotation was then based on homology searches performed with BLAST programs against the NR database, including domain searches with CD-Search and the PRIAM database [78,79,80]. Moreover, the determination of potential protein functions was also based on the searches performed on the HHpred server using HHpred or HMMER tools against the PDB_mmCIF70_11_Oct, SCOPe70_2.07, COG_KOG_v.1.0, and NCBI_Conserved_Domains (CD)_v3.16 or the nr50_1_Oct databases [81]. Putative tRNA genes were identified with the tRNAScan-SE v2.0 and ARAGORN programs [82,83].

### 3.15. Comparative Genomics

Genomes of the isolated phages were compared with prophage regions indicated in the *Ochrobactrum* spp. genomes and the phage genomes from NCBI genome browser (accessed on 10 July 2019) using protein-based similarity network constructed with vConTACT v2. During the program run, ProkaryoticViralRefSeq85-Merged was used as the reference database. The phages present in that database were removed from the set of genomes downloaded from the NCBI to avoid redundancy. In total, 10629 phage genomes were analyzed using the same settings for vConTACT as described in Section 3.11. The resulting network was filtered using a Python script and only the nodes reflecting the phages connected to (pro)phages infecting *Ochrobactrum* spp. and edges reflecting the similarity between the two phages connecting those nodes were retained. The resulting network was visualized in Gephi v0.92 using ForceAtlas 2 and Noverlap layouts to arrange the nodes in a two-dimensional space [84,85].

### 3.16. Nucleotide Sequence Accession Numbers

The nucleotide sequences of the vB_OspM_OC and vB_OspP_OH phages have been deposited in the GenBank (NCBI) database with the accession numbers MT028491 and MT028492, respectively.

## 4. Conclusions

In this study, the biological and genomic properties of two novel phages infecting *Ochrobactrum* sp. POC9, the strain enhancing the reduction of organic biomass and biogas production in WWTPs, was characterized. The identified phages, vB_OspM_OC (myovirus) and vB_OspP_OH (podovirus), are the first (to the best of our knowledge) virulent phages observed to infect *Ochrobactrum* spp. so far. The vB_OspM_OC virus was recognized as a representative of giant (jumbo) phages. Moreover, vB_OspM_OC encodes a CcrM-like DNA methyltransferase, which constitutes another example of the molecular mimicry between the phage-encoded DNA methylases and host-specific regulatory MTase in *Alphaproteobacteria*.

In this study, a thorough identification of the prophages in *Ochrobactrum* spp. genomes combined with a complex comparative genomic analysis was also performed. This analysis discovered 277 prophages within 104 out of 113 analyzed genomes of *Ochrobactrum* spp. Moreover, by applying similarity networking, it was shown that that nearly 30% of *Ochrobactrum* viruses shared homologies exclusively with other *Ochrobactrum* prophages or formed orphan nodes, which exemplifies the uniqueness of this (pro)phages. This global analysis also revealed the relatives of the vB_OspM_OC and vB_OspP_OH phages. It was shown that vB_OspM_OC is related to giant T4-like phages, while vB_OspP_OH created a separate sub-cluster with another podovirus (*Sinorhizobium meliloti* phage phiM5) and showed some similarities to the *Vibrio* and *Alteromonas* podoviruses, as well as the *Salmonella* and *Acinetobacter* siphoviruses.

Moreover, in this study, bacteriophages were recognized as another factor besides chemical (e.g., metal ions) and physical (e.g., pH or temperature) agents that influence bacterial cultures applied to environmental biotechnologies. The presence of phages may be a critical, or at least important, factor interfering in the overall effectiveness of bioaugmentation and thus the success of the whole biotechnological process.

## Figures and Tables

**Table 1:** Sizes of the heads and tails of the isolated *Ochrobactrum* phages.

Phage	Head Width (nm) *	Head Length (nm) *	Tail Width (nm) *	Tail Length (nm) *
vB_OspM_OC	97.6 ± 7.8	102.9 ± 8.0	28.9 ± 2.9	68.3 ± 11.4
vB_OspP_OH	55.1 ± 1.6	55.8 ± 1.9	11.5 ± 1.6	7.6 ± 0.9

* The presented sizes are the averages (± standard deviation) calculated from measurements of 10 randomly picked phage particles.

## Supplementary Materials

Supplementary materials can be found at https://www.mdpi.com/1422-0067/21/6/2096/s1. Figure S1: Multisequence alignment of CcrM and CcrM-like DNA methyltransferases. Figure S2: Genomic comparison of *Sinorhizobium* phage phiM5 and vB_OspP_OH. Figure S3: One-step growth curves of the vB_OspM_OC phage. Figure S4: Bacterial culture collapse as the effect of phage infection. Table S1: The comparison of vB_OspM_OC phage-encoded proteins with T4 and T4-like phages using protein BLAST (1e-5 e-value threshold). Table S2: Prophages identified within *Ochrobactrum* spp. genomes.
