# Supplementary material for: Identification and Characterization of the First Virulent Phages, Including a Novel Jumbo Virus, Infecting Ochrobactrum spp
Source: Int J Mol Sci. 2020 Mar 18;21(6):2096. doi: 10.3390/ijms21062096 (PMC7139368; doi:10.3390/ijms21062096)
Supplement: Supplementary file 1 [file ijms-21-02096-s001.pdf]

Table S1. The comparison of vB_OspM_OC phage-encoded proteins with T4 and T4-like phages using protein BLAST (1e-5 e-value threshold). In case of multiple hits for single proteins, only the one with higher query coverage per HSP was considered. Within a table, a color scale was used to reflect the percentage of protein sequence identity between vB_OspM_OC proteins and those from other phages. Where any homologous protein was identified, cells were left blank.

	Proteobacteria																					
	**γ**										**β**		**α**									
**vB_OspM_OC**	Escherichia_phage_T4_AF158101	Aeromonas_phage_44RR2,8t_AY375531	Aeromonas_phage_Aeh1_AY266303	Escherichia_phage_RB43_AY967407	Escherichia_phage_vB_EcoM_ACG-C40_JN986846	Vibrio_phage_KVP40_AY283928	Pseudomonas_phage_pf16_KU873925,1	Stenotrophomonas_phage_IME-SM1_KR560069,1	Stenotrophomonas_phage_YB07_MK580972,1	Stenotrophomonas_phage_vB_SmaS-DLP_6_KU682439,2	Acidovorax_phage_ACP17_KY979132,2	Delftia_phage_PhiW-14_GQ357915	Agrobacterium_phage_Atu_ph04_MF403007,1	Agrobacterium_phage_Atu_ph07_MF403008,1	Caulobacter_phage_Cr30_KF301602,1	Pelagibacter_phage_HTVC008M_KC465899,1	Rhizobium_phage_vB_RleM_P10VF_KM199770,1	Sinorhizobium_phage_phiM19_KR052481,1	Sinorhizobium_phage_phiM7_KR052480,1	Sinorhizobium_phage_phiM9_KP881232,1	Sinorhizobium_phage_phiN3_KR052482,1	Sphingomonas_phage_PAU_JQ362498,1
PhiOC_p001	37	34	36	36	37	35	37	36	36	41	36	31		28	38	39	30	36	36	32	36	
PhiOC_p002 UvsX RecA-like recombination protein	30		30	30	30	32	27	31	31	32	32	26	26	29	31	52	27	33	33	27	33	
PhiOC_p004 DNA-directed DNA polymerase	34	32	34	33	34	34	34	34	34	34	33	30	30	26	36	38	31	36	36	31	36	25
PhiOC_p007 translation regulatory protein RegA	50	48	48	44	50	48	46	41	41	55	40	38			44	55		59	59		59	
PhiOC_p008 DNA polymerase sliding clamp loader subunit	26			28		27	33	34	34	28	36	30			32	31		29	29	30	29	
PhiOC_p009 DNA polymerase sliding clamp loader subunit	31	31	32	32	31	39	40	35	35	44	37	35	33	30	37	42	36	39	39	31	39	36
PhiOC_p010 DNA polymerase sliding clamp			21			26	26	26	25	29	27	22			24	23		23	23		23	

	**unknown**	**Terrabacteria**																							
**vB_OspM_OC**	Lake_Baikal_phage_Baikal-20-5m-C28_MG198570,1	Cyanophage_P-RSM1_HQ634175,1	Cyanophage_P-RSM3_HQ634176,1	Cyanophage_S-RIM14_KX349305,1	Cyanophage_S-RIM14_KX349306,1	Cyanophage_S-RIM32_KU594606,1	Cyanophage_S-SSM6a_HQ317391,1	Cyanophage_S-SSM6b_HQ316603,1	Prochlorococcus_phage_MED4-213_HQ634174,1	Prochlorococcus_phage_P-SSM7_GU071103,1	Prochlorococcus_phage_P-TIM68_KM359505,1	Synechoccus_phage_S-B05_MK799832,1	Synechococcus_phage_ACG-2014f_KJ019141,1	Synechococcus_phage_S-B68_MK016664,1	Synechococcus_phage_S-CAM22_KU686207,1	Synechococcus_phage_S-CAM7_KU686212,1	Synechococcus_phage_S-CAM9_KU686204,1	Synechococcus_phage_S-CRM01_HQ615693,1	Synechococcus_phage_S-IOM18_HQ317383,1	Synechococcus_phage_S-PM2_AJ630128,1	Synechococcus_phage_S-SKS1_HQ633071,1	Synechococcus_phage_S-SSM7_GU071098,1	Synechococcus_phage_S-ShM2_GU071096,1	Synechococcus_phage_metaG-MbCM1_JN371769,1	Synechococcus_virus_S-PRM1_MH629685,1
PhiOC_p001	32	40	38	38	38	38	37	36	40	43	40	39	40	35	42	39	39	37	41	37	37	37	38	39	40
PhiOC_p002 UvsX RecA-like recombination protein	30	49	50	48	48	49	50	47	47	49	47	46	51	51	49	47	48	51	49	49	47	50	48	64	49
PhiOC_p004 DNA-directed DNA polymerase	36	38	39	40	40	38	37	40	38	39	38	39	41	37	41	37	37	38		40	39	37	40	45	37
PhiOC_p007 translation regulatory protein RegA	55	55	56	51	51	56	54	57	55	53	55	54	55	55	54	51	58	58	54	56	54	54	51	54	59
PhiOC_p008 DNA polymerase sliding clamp loader subunit	31	29	33	35	35	34	28	31	32	35	25	30	31	35	35		32	36	33	32	25	28	31	33	31
PhiOC_p009 DNA polymerase sliding clamp loader subunit	39	39	40	39	39	38	40	39	38	40	39		40	37	40	40	38	39	41	41	41	40	39	39	37
PhiOC_p010 DNA polymerase sliding clamp	27	25	25	24	24	24	24	22		22	26	26	24	26	22	26	27	24	24	25	27	24	24	23	27

PhiOC_p011 hypothetical protein																33								39	37	36	36	43	37	39	38	35	34	35	41		37	40	35		37		43	37	36	40	36
PhiOC_p012 putative glycosyltransferase										52					50			45	45		45														29												
PhiOC_p013 putative cobalt chelatase subunit CobS										35						38							37	36	38	36	36	39	36	37	36	37	38	39	38		36	39	37	42	37	38		36	36	39	37
PhiOC_p014 cobalamin biosynthesis protein CobT										32						25							27	30	28	29	29	24	28	29	20	30	28	26	26		30	26	24	29	23	27		28	29	27	25
PhiOC_p015 peptide deformylase										32					29																				30			29						32			
PhiOC_p016 phage endonuclease subunit 2	32	33	42	33	32	43	31	32	32	35	36	27	30	26	32	37	30	35	35	27	35		34	34	35	36	36	36	35	36	35	34	35	35	36	33	33	35	36	34	33	37	35	35	36	34	36
PhiOC_p017 phage endonuclease subunit 1	30	27	29	27	30	32	27	30	30	37	30	24		30	29	32		30	30		30		30	30	29	29	29	32	29	29	31	29	33	27	34	29	28	35	31	26	27	29	33	29	29	31	31
PhiOC_p020 RNA polymerase sigma factor for late transcription	28	25	28	25	28	37	28			28		28			26	34		36	36		34		33	35	34	35	35	38	33	33	32	32	34	31	33	37	31	35	39	39	29	40	44	33	35	35	39
PhiOC_p021 UvsW ATP-dependent DNA helicase	34	34	34	33	34	34	32	39	39	39	34	32	32	27	35	37	33	36	36	35	36	29	40	39	37	38	38	39	38	38	37	38	38	39	38	39	40	38	37	37	40	40	39	39	38	39	37
PhiOC_p022 UvsY protein							25	26	26	29	26		28		33	29	31	33	33		31		25	32	31	32	32	24	32	31	29	30	31	31	28	29	30	27	27	28	32	25	27	32	32	30	27
PhiOC_p028 baseplate wedge subunit						26				26	20	23			25	25	23	24	24	26	25		23	24	24	28	28		32	28	25	22	29	30	22	28	25	23	27	25	25		29	32	28	25	28
PhiOC_p029 GIY-YIG domain-containing protein											32										38																										
PhiOC_p030 major capsid protein	33	33	33	35	33	37	48	46	46	55	42	44	34	27	50	51	35	53	53	34	53		51	47	48	47	47	49	52	48	51	46	53	51	52	51	48	50	56	49	48	50	53	52	47	48	53
PhiOC_p031 prohead core protein	31	29	30	30	31	26	27			35					33	36		31	31		31		30	37	34	39	39	39	34	36	35	35	35	36	37	37	38	38	39	37	38	41	35	34	39	37	40
PhiOC_p032 prohead core scaffolding protein and protease	35	37	35	40	35	41	36	36	35	40	37	36	41	37	35	43	43	43	43	45	43		41	41	40	53	53	46	46	44	47	42	44	43	48	42	42	49	46	49	40	46	45	46	53	43	46
PhiOC_p034 portal protein	39	39	38	37	39	39	36	37	38	45	34	34	34	36	43	45	35	45	45	34	45	21	47	48	49	46	46	43	42	47	45	48	44	43	42	41	49	43	43	40	49	43	45	42	46	49	45
PhiOC_p035 baseplate wedge subunit	36	35	36	35	36	32	37	26	26	46	33	25			42	30	25	43	43	25	43		38	27	33	26	26	33	35	32	30	29	30	31	33	32	31	37	33	35	30	30	36	35	26	30	32
PhiOC_p036 tail connector/sheath protein	44	44	45	31	44	33	41	36	36	47	47	34	37	32	42	45	38	45	45	40	44		51	42	36	34	34	37	37	42	38	41	35	43	34	45	41	41	40	45	43	41	35	37	41	40	40
PhiOC_p037 terminase small subunit										31					25			29	29		29																										
PhiOC_p038 tail terminator	27	30	32	27	27	33	29	29	29	34	28	22	26	29	37	31	27	33	33	29	33		29	34	32	36	36	34	36	31	27	35	34	32	31	34	34	31	33	30	34	35		36	36	36	33
PhiOC_p039 neck protein	26	25	29	30	25	26	31		26	36	28	27	32		37	30	23	34	34		35		33	35	34	33	33	35	33	37	34	35	38		38	34	35	40	37	32	33	28	34	33	33	35	39
PhiOC_p043 neck protein	24	30	45	22	24	25	29	29	29	39	32	25	26		37	33	28	34	34	27	34		33	28	29	28	28	31	32	27	31	29	35	30	43	37	29	27	38	39	29	30	33	32	28	30	39
PhiOC_p045 hypothetical protein										50																																					
PhiOC_p046 tail fiber receptor recognition protein			35	37		44							36			32	38			39			42						33						36			47	49					36			51
PhiOC_p048 hypothetical protein							34	32	31	39	30	27	30		36	33	31	31	31	33	31		36	32	31	33	33	30	35	33	33	31	33	35	33	33	31	33	34	45	29	31		35	33	31	
PhiOC_p049 phage baseplate structural protein							29	32		25	28				35	35	29	30	29	29	30		27	36	36	27	27	26	25	32	32	28	34	32	31	33	26	24	35	31	29	28	25	25	27	26	
PhiOC_p050 phage baseplate wedge subunit				24			53	26	26	35	24				47	36	24	41	41	25	41		38	30	41	33	33			30	30	29			49	24	44			47	33	43			33	29	
PhiOC_p051 phage baseplate wedge subunit	29	29	29	29	29	27	28	27	27	31	27	27	26		29	35	27	30	30	29	30	28	31	30	31	31	31	33	30	32	31	32	32	32	28	31	32	31	31	33	34	31	33	30	31	31	
PhiOC_p052 phage baseplate wedge subunit			29			33	34		31	36	29	33			33	30		40	40		40		26	38	24	28	28	32	28	29	33	30	34	31	30	28	30	24	28	32	34	32	28	28	28	37	
PhiOC_p054 DprA-like DNA processing protein						44				39		33					37			40																											

PhiOC_p057 hypothetical protein						36								35							54																										
PhiOC_p063 baseplate hub subunit and tail lysozyme	46	44	47	41	46	42	45	52	52	44	46	27	55	37	53	43	51	51	51	46	51		57	46	46	48	48	40	37	50	42	49	37	37		46	49			49	48	37	35	37	48	46	
PhiOC_p064 baseplate hub subunit and tail lysozyme										25		23			25	24	20	29	29		29		29																								
PhiOC_p065 baseplate wedge protein	26	26	33		26	29	28	30	30	33	29	30			29	28		28	28	23	28		31	30	30	31	31	32	33	32	28	30	30	30	26	36	31	22	25	29	31	30	27	33	31	32	24
PhiOC_p066 baseplate tail tube cap							25	26	27	26	27	29			21	30	26	25	25	27	25		24	22				27	27	24	28	26	25	27	23	26	24	26	27	23	24	30	28	27			28
PhiOC_p067 head completion protein	36	38	38	37	36	36	48	35	35	39	39	37	41	34	34	46	40	43	43	42	43	34	41	44	44	43	43	42	39	46	39	45	41	45	45	38	43	46	43	39	42	43	47	39	43	43	44
PhiOC_p068 DNA end protector protein	29	32	32	32	29		36	41	41	34	35	33	30		33	34	32	39	39	30	39		34					27	27										29			26		27			29
PhiOC_p069 hypothetical protein						23	21			26		20			26	24	25			27																											
PhiOC_p070 baseplate wedge protein			24			43	31	30	31	36	27	25			34	32		30	30	25	30		34	34	35	34	34	36	34	35	31	35	33	34	30	31	34	23	32	30	33	33	32	34	34	33	30
PhiOC_p071 hypothetical protein																										41	41	46			44		39		44			46				43	39		41		
PhiOC_p073 phage terminase large subunit	35	36	34	34	34	38	38	37	37	40	37	32	34	29	40	44	34	36	36	35	36	27	34	40	40	42	42	41	41	40	40	40	42	43	42	41	42	41	42	43	41	41	43	41	45	40	42
PhiOC_p083 single-stranded DNA-binding protein	33	34	31	29	33	37	30	32	32	34	29	26	24		34	34	27	32	32	29	32		33	38	34	33	33	34	33	35	37	35	34	38	34	35	36	36	36	36	39	34	38	33	33		36
PhiOC_p085 T4-like RNA polymerase-associated protein Gp33																												35					36		36					30		34					
PhiOC_p087 PhoH-like protein				29		32	30			48	42	30	31	30	44	43	31	44	44	27	44		39	41	43	41	41	41	42	41	44	40	44	42	36	37	44	35	44	40	41	42		42	41	43	44
PhiOC_p088 putative Com-like regulatory protein										41	40				37	37		37	37		37									50	33			36								32					
PhiOC_p090 T4-like DNA ligase										31					22	27		29	29		29										27	26		25													27
PhiOC_p091 T4-like Ribonuclease H	28	29	31	26	28	28		35	34	30	31		29		33	29	28	33	33	25	33		31					31								31				27		31					
PhiOC_p095 hypothetical protein								29	29	30	30				26			28	28		28		32																								
PhiOC_p099 glycosyltransferase family 1 protein										42					33			36	36		36																										
PhiOC_p104 metallophosphatase domain-containing protein								31	30		26				28						31																										
PhiOC_p106 polinucleotide kinase															33						34																										
PhiOC_p107 T4-like RNA ligase 1	31	30	27	29	30	27		31	32					30	33			33	33		33																										
PhiOC_p108 putative response regulator protein										33				48																													32				
PhiOC_p113 hypothetical protein														44							33																										
PhiOC_p116 HD superfamily hydrolase								35	35		41		37																																		
PhiOC_p117 putative DNA replication protein														45																																	
PhiOC_p118 nicotinamide-nucleotide adenylyltransferase NadR														39				44	44		44																										
PhiOC_p119 nicotinamide mononucleotide transporter PnuC						27								33				36	36		36																										
PhiOC_p127 nicotinate phosphoribosyltransferase NadV		42	40	42		44	30	42	42	45	48							49	43		43																										
PhiOC_p129 hypothetical protein																		62	62		62																										
PhiOC_p131 bifunctional NMN adenylyltransferase/Nudix hydrolase		28	30	31		32		32	32	33	27							39	39		39																										

PhiOC_p135 serine/threonine protein phosphatase																		30	31		30																										
PhiOC_p137 HNH endonuclease				38														45	45															33													
PhiOC_p139 putative nucleotidyltransferase	26	26	28	27	26	24								24	25			28	28		28																										
PhiOC_p142 hypothetical protein																		48	48		48																										
PhiOC_p144 lytic transglycosylase														67																																	
PhiOC_p146 chitinase												41			41			37	37		45		39													25											
PhiOC_p152 putative Ro sixty-related protein			38			39				44								38	38		38																										
PhiOC_p163 HNH endonuclease												37		34				40	40		37														37	41		35		42			42				
PhiOC_p165 carbohydrate-binding protein								28	27				26																									33									
PhiOC_p166 thymidine kinase	40	43	41	39	40	39								23																																	
PhiOC_p175 hypothetical protein														34																																	
PhiOC_p178 deoxycytidylate deaminase	46	34	28		46	36	27			34			39		29		43			42		36	44																								
PhiOC_p184 NADAR family protein							44	42				46																																			
PhiOC_p187 HflK-like protein										35																																					
PhiOC_p195 hypothetical protein		24				34																																									
PhiOC_p201 DNA-binding protein															48		52			52																											
PhiOC_p205 DNA ligase															40			36	36		36																			25							
PhiOC_p212 N6-adenine specific DNA methyltransferase														27											24			25		40	43	46	40				28	28					45				
PhiOC_p228 hypothetical protein														41																																	
PhiOC_p229 putative glutamyl-tRNA(Gln) amidotransferase subunit A						31	34											35	35		35																										
PhiOC_p235 glutathionylspermidine synthase family protein		36																																													
PhiOC_p237 hypothetical protein		26																																													
PhiOC_p243 hypothetical protein																				57																											
PhiOC_p245 putative anti-TRAP protein				63										66																																	
PhiOC_p248 HNH endonuclease					30									48																										37							
PhiOC_p251 hypothetical protein																		31	31		31																										
PhiOC_p252 HNH endonuclease				33														41	41															37													
PhiOC_p267 hypothetical protein															27																																
PhiOC_p291 hypothetical protein														38																																	
PhiOC_p292 hypothetical protein														45																																	
PhiOC_p301 tail fiber domain-containing protein	36				38																												36					32									41
PhiOC_p302 putative tail receptor-binding protein								30																																							

PhiOC_p313 hypothetical protein			37														40	29	29																												
PhiOC_p320 outer membrane protein														32																																	
PhiOC_p321 putative endonuclease segX	58			36	56	33	37				43				51		33	34	34	46	34																										
PhiOC_p322 metallophosphatase														45				54	54		52																										
PhiOC_p324 hypothetical protein								41	42		32							38	38		40		38	45	44	43	43		44	51	46	46	47	43	44			45	42		47		47	44	43		
PhiOC_p332 putative N-acetylmuramoyl-L-alanine amidase														30																																	
PhiOC_p333 c1q globular head like domain containing protein												38											30																								
PhiOC_p334 phage tail fiber protein		35	25	26	25	24										33							33						31						38			39	40					31			40
PhiOC_p335 putative glycosylase								37	33				31																																		
PhiOC_p336 hypothetical protein			35	33																45																											
PhiOC_p337 putative glycosylase								32	29																																						
PhiOC_p344 putative D-alanine D-alanine ligase														29							23																			28							
PhiOC_p346 putative quorum-sensing-regulated virulence factor				34													41	41	41	45	41																										
PhiOC_p355 ribonucleoside-diphosphate reductase subunit beta						21	22									25						42																									
PhiOC_p356 ribonucleotide-diphosphate reductase subunit alpha	25		29	25	26	25	24	25	25	24	23	21	22	23	22	25	24			24		47	24	23	23	23	23	26	23	26	24	25	24	23	26	26	24	24	24	25	24	25	24	23	23	24	24
PhiOC_p357 ribonucleotide reductase stimulatory protein																						35																									
PhiOC_p358 glutaredoxin						34				33		38			33								32																								
PhiOC_p359 flavin-dependent thymidylate synthase											36			27									28	27	29	27	27	27	26	26	29	28	27	27		27	26		28	28	25	28	27	26	27	26	28
PhiOC_p360 DNA primase	31	31	32	31	31	29	33	27	27	32	28	29	29		29	31	26	31	31	29	31			35	34	34	34	36	35	34	35	35	32	36	35	35	34	35	36	36	35	35	34	34	34	39	34
PhiOC_p363 hypothetical protein																		30	30		30																										
PhiOC_p368 hypothetical protein																		47	47		51																										
PhiOC_p369 DNA methyltransferase, NPPF				47														62	62		63																										
PhiOC_p370 methyltransferase, FkbM family										47								45			42																										
PhiOC_p374 hypothetical protein														57				58	58		58																										
PhiOC_p377 hypothetical protein							40			40					45	35		37	37		37		37	38	36	35	35		34	30	28	31	28	31	31	31	36	29		31	36	38	36	34	35		33
PhiOC_p378 hypothetical protein															40																																
PhiOC_p380 hypothetical protein																													39															39			
PhiOC_p381 hypothetical protein			38				31	34	34																																						
PhiOC_p383 RNA 2',3'-cyclic phosphodiesterase /2'-5' RNA ligase LigT																		38	38		38																										
PhiOC_p385 hypothetical protein																				29																											
PhiOC_p386 putative rIII lysis inhibition accessory protein, rapid lysis phenotype										38						34		40	40		41		29																								

PhiOC_p388 metallophosphatase domain-containing protein	28
PhiOC_p389 ImmA/IrrE family metallopeptidase	25

Table S2. Prophages identified within *Ochrobactrum* spp. genomes.

Prophages and their hosts																
#	prophage name	prophage size [bp]	CDS	tRNA	Host name	Host strain	Host replicon accession number	Prophage region start	Prophage region stop	Host genome assembly	Host genome completeness level	Host genome size (Mb)	Host GC%	Host scaffolds	Host CDS
1	vB_OspX_pp1	48017	76	0	*O. anthropi* ATCC 49188	ATCC 49188	NC_009667.1	224589	272605	GCA_000017405.1	Complete	5.20578	56.1453	6	4808
2	vB_OspX_pp2	13636	19	0	*O. anthropi* ATCC 49188	ATCC 49188	NC_009667.1	918675	932310	GCA_000017405.1	Complete	5.20578	56.1453	6	4808
3	vB_OspX_pp3	66203	98	1	*O. anthropi* ATCC 49188	ATCC 49188	NC_009667.1	1574190	1640392	GCA_000017405.1	Complete	5.20578	56.1453	6	4808
4	vB_OspX_pp4	42622	56	1	*O. anthropi* ATCC 49188	ATCC 49188	NC_009667.1	2408860	2451481	GCA_000017405.1	Complete	5.20578	56.1453	6	4808
5	vB_OspX_pp5	35856	48	0	*O. intermedium* LMG 3301	LMG 3301	NZ_ACQA01000001.1	1801130	1836985	GCA_000182645.1	Scaffold	4.72539	57.7	4	4235
6	vB_OspX_pp6	30859	38	1	*O. intermedium* LMG 3301	LMG 3301	NZ_ACQA01000002.1	1073630	1104488	GCA_000182645.1	Scaffold	4.72539	57.7	4	4235
7	vB_OspX_pp7	36900	57	2	*O. intermedium* LMG 3301	LMG 3301	NZ_ACQA01000004.1	17005	53904	GCA_000182645.1	Scaffold	4.72539	57.7	4	4235
8	vB_OspX_pp8	38669	49	0	*O. anthropi* CTS-325	CTS-325	NZ_JH605226.1	693215	731883	GCA_000251205.1	Scaffold	4.72698	56	15	4350
9	vB_OspX_pp9	45427	72	0	*O. anthropi* CTS-325	CTS-325	NZ_JH605228.1	902261	947687	GCA_000251205.1	Scaffold	4.72698	56	15	4350
10	vB_OspX_pp10	15013	20	0	*O. intermedium* M86	M86	NZ_AOGE01000001.1	2095	17107	GCA_000332835.1	Contig	5.18869	57.9	148	4793
11	vB_OspX_pp11	13521	23	0	*O. intermedium* M86	M86	NZ_AOGE01000005.1	1	13521	GCA_000332835.1	Contig	5.18869	57.9	148	4793
12	vB_OspX_pp12	53033	64	0	*O. intermedium* M86	M86	NZ_AOGE01000011.1	56118	109150	GCA_000332835.1	Contig	5.18869	57.9	148	4793
13	vB_OspX_pp13	29217	40	0	*O. intermedium* M86	M86	NZ_AOGE01000027.1	1	29217	GCA_000332835.1	Contig	5.18869	57.9	148	4793
14	vB_OspX_pp14	31190	44	1	*O. intermedium* M86	M86	NZ_AOGE01000038.1	9067	40256	GCA_000332835.1	Contig	5.18869	57.9	148	4793
15	vB_OspX_pp15	41997	46	0	*O. intermedium* M86	M86	NZ_AOGE01000048.1	1	41997	GCA_000332835.1	Contig	5.18869	57.9	148	4793
16	vB_OspX_pp16	49149	77	0	*Ochrobactrum* sp. CDB2	CDB2	NZ_AKVI01000072.1	118797	167945	GCA_000344725.1	Contig	4.97123	53.6	146	4503
17	vB_OspX_pp17	63014	86	2	*Ochrobactrum* sp. EGD-AQ16	EGD-AQ16	NZ_AWEU01000034.1	284869	347882	GCA_000465835.2	Contig	4.83434	57.6	53	4465
18	vB_OspX_pp18	19202	30	0	*O. intermedium* 229E	229E	NZ_ASXJ01000363.1	6652	25853	GCA_000472165.1	Contig	4.80822	57.9	378	3260
19	vB_OspX_pp19	29840	41	0	*O. rhizosphaerae* SJY1	SJY1	NZ_AZRT01000012.1	33626	63465	GCA_000559065.1	Contig	5.24577	54.2	198	4873
20	vB_OspX_pp20	6645	12	0	*O. rhizosphaerae* SJY1	SJY1	NZ_AZRT01000048.1	76555	83199	GCA_000559065.1	Contig	5.24577	54.2	198	4873
21	vB_OspX_pp21	28985	42	0	*O. rhizosphaerae* SJY1	SJY1	NZ_AZRT01000050.1	195	29179	GCA_000559065.1	Contig	5.24577	54.2	198	4873
22	vB_OspX_pp22	27694	38	0	*O. rhizosphaerae* SJY1	SJY1	NZ_AZRT01000082.1	300129	327822	GCA_000559065.1	Contig	5.24577	54.2	198	4873
23	vB_OspX_pp23	36034	45	0	*O. rhizosphaerae* SJY1	SJY1	NZ_AZRT01000188.1	198314	234347	GCA_000559065.1	Contig	5.24577	54.2	198	4873
24	vB_OspX_pp24	38387	49	0	*O. anthropi*	W13P3	NZ_KK073936.1	256577	294963	GCA_000585655.1	Scaffold	5.27782	56.3	43	4947
25	vB_OspX_pp25	43329	47	0	*O. anthropi*	W13P3	NZ_KK073937.1	598833	642161	GCA_000585655.1	Scaffold	5.27782	56.3	43	4947
26	vB_OspX_pp26	48335	80	0	*O. anthropi*	W13P3	NZ_KK073938.1	225382	273716	GCA_000585655.1	Scaffold	5.27782	56.3	43	4947
27	vB_OspX_pp27	26082	30	0	*O. anthropi*	W13P3	NZ_KK073938.1	532607	558688	GCA_000585655.1	Scaffold	5.27782	56.3	43	4947
28	vB_OspX_pp28	12860	20	0	*O. anthropi*	W13P3	NZ_KK073942.1	189870	202729	GCA_000585655.1	Scaffold	5.27782	56.3	43	4947

29	vB_OspX_pp29	16473	22	0	*O. anthropi*	W13P3	NZ_KK073943.1	144894	161366	GCA_000585655.1	Scaffold	5.27782	56.3	43	4947
30	vB_OspX_pp30	48309	59	0	*O. intermedium* 2745-2	2745-2	NZ_JFHY01000011.1	286276	334584	GCA_000612245.1	Contig	4.80018	57.6	95	4430
31	vB_OspX_pp31	44184	55	2	*O. intermedium* 2745-2	2745-2	NZ_JFHY01000013.1	349227	393410	GCA_000612245.1	Contig	4.80018	57.6	95	4430
32	vB_OspX_pp32	15910	18	0	*O. intermedium* 2745-2	2745-2	NZ_JFHY01000042.1	100323	116232	GCA_000612245.1	Contig	4.80018	57.6	95	4430
33	vB_OspX_pp33	24806	40	0	*O. intermedium* 2745-2	2745-2	NZ_JFHY01000056.1	105917	130722	GCA_000612245.1	Contig	4.80018	57.6	95	4430
34	vB_OspX_pp34	11763	22	0	*Ochrobactrum* sp. UNC390CL2Tsu3S39	UNC390CL2Tsu3S39	NZ_JOOD01000004.1	95	11857	GCA_000712085.1	Contig	4.62463	56	16	4315
35	vB_OspX_pp35	62469	86	0	*Ochrobactrum* sp. UNC390CL2Tsu3S39	UNC390CL2Tsu3S39	NZ_JOOD01000009.1	251900	314368	GCA_000712085.1	Contig	4.62463	56	16	4315
36	vB_OspX_pp36	13698	19	0	*O. anthropi*	OAB	NZ_CP008820.1	450813	464510	GCA_000742955.1	Complete	4.90116	56.0831	4	4472
37	vB_OspX_pp37	33807	46	0	*O. anthropi*	ML7	NZ_JYFX01000027.1	42239	76045	GCA_000878465.1	Scaffold	4.90418	56	74	4554
38	vB_OspX_pp38	89397	122	0	*O. anthropi*	ML7	NZ_JYFX01000033.1	126545	215941	GCA_000878465.1	Scaffold	4.90418	56	74	4554
39	vB_OspX_pp39	17608	21	0	*O. anthropi*	ML7	NZ_JYFX01000033.1	292958	310565	GCA_000878465.1	Scaffold	4.90418	56	74	4554
40	vB_OspX_pp40	46868	63	1	*O. anthropi*	ML7	NZ_JYFX01000057.1	197267	244134	GCA_000878465.1	Scaffold	4.90418	56	74	4554
41	vB_OspX_pp41	23997	29	0	*O. anthropi*	FRAF13	NZ_LSVB01000013.1	48552	72548	GCA_001575075.1	Contig	4.53807	56	17	4141
42	vB_OspX_pp42	34820	53	2	*O. intermedium*	T	NZ_LXEK01000046.1	18	34837	GCA_001637305.1	Contig	3.9389	57.8	48	3645
43	vB_OspX_pp43	51713	75	1	*O. intermedium*	KCJK1738	NZ_LXPU01000022.1	89760	141472	GCA_001641495.1	Contig	4.70359	57.7	47	4393
44	vB_OspX_pp44	32626	42	0	*O. intermedium*	KCJK1738	NZ_LXPU01000043.1	137881	170506	GCA_001641495.1	Contig	4.70359	57.7	47	4393
45	vB_OspX_pp45	14649	18	0	*O. pseudogrignonense*	K8	NZ_CP015775.1	538897	553545	GCA_001652485.1	Complete	4.9928	53.7131	2	4577
46	vB_OspX_pp46	41961	46	0	*O. pseudogrignonense*	K8	NZ_CP015775.1	3288698	3330658	GCA_001652485.1	Complete	4.9928	53.7131	2	4577
47	vB_OspX_pp47	17407	27	1	*O. cytisi*	IPA7.2	NZ_MOEC01000018.1	130	17536	GCA_001876955.1	Scaffold	5.96536	55.4	192	5419
48	vB_OspX_pp48	15213	28	2	*O. intermedium*	SA148	NZ_LWEA01000008.1	1	15213	GCA_001917355.1	Contig	4.9132	57.6	38	4492
49	vB_OspX_pp49	46413	69	0	*O. intermedium*	SA148	NZ_LWEA01000010.1	128685	175097	GCA_001917355.1	Contig	4.9132	57.6	38	4492
50	vB_OspX_pp50	36830	57	0	*Ochrobactrum* sp. P6BS-III	P6BS-III	NZ_MPPJ01000002.1	164333	201162	GCA_002016635.1	Contig	5.25313	56.6	65	4644
51	vB_OspX_pp51	32992	44	0	*O. pituitosum*	AA2	NZ_CP018780.1	1069957	1102948	GCA_002025625.1	Complete	5.46737	53.5989	4	4864
52	vB_OspX_pp52	39339	58	0	*O. pituitosum*	AA2	NZ_CP018782.1	60279	99617	GCA_002025625.1	Complete	5.46737	53.5989	4	4864
53	vB_OspX_pp53	28270	31	0	*O. thiophenivorans*	DSM 7216	NZ_NNRJ01000019.1	224417	252686	GCA_002252445.1	Contig	4.36478	51.6	77	3943
54	vB_OspX_pp54	15651	21	0	*O. thiophenivorans*	DSM 7216	NZ_NNRJ01000054.1	44336	59986	GCA_002252445.1	Contig	4.36478	51.6	77	3943
55	vB_OspX_pp55	21540	21	0	*O. rhizosphaerae*	PR17	NZ_NNRK01000022.1	190012	211551	GCA_002252475.1	Contig	4.90401	53	36	4470
56	vB_OspX_pp56	53760	69	2	*O. rhizosphaerae*	PR17	NZ_NNRK01000025.1	209363	263122	GCA_002252475.1	Contig	4.90401	53	36	4470
57	vB_OspX_pp57	20790	22	0	*O. rhizosphaerae*	PR17	NZ_NNRK01000026.1	229224	250013	GCA_002252475.1	Contig	4.90401	53	36	4470
58	vB_OspX_pp58	17653	23	0	*O. rhizosphaerae*	PR17	NZ_NNRK01000026.1	446909	464561	GCA_002252475.1	Contig	4.90401	53	36	4470
59	vB_OspX_pp59	34953	42	0	*O. rhizosphaerae*	PR17	NZ_NNRK01000033.1	56512	91464	GCA_002252475.1	Contig	4.90401	53	36	4470
60	vB_OspX_pp60	20408	24	1	*O. grignonense*	OgA9a	NZ_NNRL01000163.1	457709	478116	GCA_002252505.1	Contig	4.83827	54.1	169	4349
61	vB_OspX_pp61	22784	26	0	*O. grignonense*	OgA9a	NZ_NNRL01000163.1	784344	807127	GCA_002252505.1	Contig	4.83827	54.1	169	4349

62	vB_OspX_pp62	41803	63	0	*O. pseudogrignonense*	CCUG 30717	NZ_NNRM01000021.1	113573	155375	GCA_002252525.1	Contig	5.53156	54	53	5027
63	vB_OspX_pp63	15757	21	0	*O. pseudogrignonense*	CCUG 30717	NZ_NNRM01000047.1	13121	28877	GCA_002252525.1	Contig	5.53156	54	53	5027
64	vB_OspX_pp64	38683	56	0	*O. lupini*	LUP21	NZ_NNRN01000055.1	18	38700	GCA_002252535.1	Contig	5.58281	56.3	65	5150
65	vB_OspX_pp65	43458	54	0	*O. quorumnocens*	A44	NZ_CP022604.1	990825	1034282	GCA_002278035.1	Complete	5.64529	53.1734	4	5137
66	vB_OspX_pp66	43210	59	0	*O. quorumnocens*	A44	NZ_CP022604.1	1543886	1587095	GCA_002278035.1	Complete	5.64529	53.1734	4	5137
67	vB_OspX_pp67	63064	83	0	*O. pituitosum*	BU72	NZ_PHRE01000001.1	802970	866033	GCA_002803535.1	Scaffold	4.88541	53.4	9	4460
68	vB_OspX_pp68	23191	30	1	*O. pituitosum*	BU72	NZ_PHRE01000004.1	1715574	1738764	GCA_002803535.1	Scaffold	4.88541	53.4	9	4460
69	vB_OspX_pp69	47241	58	0	*Ochrobactrum* sp. 721/2009	721/2009	NZ_PCQQ01000001.1	869315	916555	GCA_002808025.1	Contig	4.93858	57.8	18	4565
70	vB_OspX_pp70	60109	86	0	*Ochrobactrum* sp. 721/2009	721/2009	NZ_PCQQ01000005.1	251862	311970	GCA_002808025.1	Contig	4.93858	57.8	18	4565
71	vB_OspX_pp71	32020	46	0	*Ochrobactrum* sp. 721/2009	721/2009	NZ_PCQQ01000007.1	32	32051	GCA_002808025.1	Contig	4.93858	57.8	18	4565
72	vB_OspX_pp72	26500	33	0	*Ochrobactrum* sp. 720/2009	720/2009	NZ_PCQR01000001.1	176855	203354	GCA_002808495.1	Contig	4.93686	57.8	18	4566
73	vB_OspX_pp73	38062	56	0	*Ochrobactrum* sp. 720/2009	720/2009	NZ_PCQR01000002.1	1044496	1082557	GCA_002808495.1	Contig	4.93686	57.8	18	4566
74	vB_OspX_pp74	53042	76	0	*Ochrobactrum* sp. 720/2009	720/2009	NZ_PCQR01000010.1	251025	304066	GCA_002808495.1	Contig	4.93686	57.8	18	4566
75	vB_OspX_pp75	35863	54	0	*Ochrobactrum* sp. 715/2009	715/2009	NZ_PCQS01000001.1	1275294	1311156	GCA_002808515.1	Contig	4.93667	57.8	18	4565
76	vB_OspX_pp76	52771	75	0	*Ochrobactrum* sp. 715/2009	715/2009	NZ_PCQS01000002.1	251531	304301	GCA_002808515.1	Contig	4.93667	57.8	18	4565
77	vB_OspX_pp77	27221	38	0	*Ochrobactrum* sp. 715/2009	715/2009	NZ_PCQS01000016.1	109501	136721	GCA_002808515.1	Contig	4.93667	57.8	18	4565
78	vB_OspX_pp78	60348	89	0	*Ochrobactrum* sp. 695/2009	695/2009	NZ_PCQT01000002.1	347059	407406	GCA_002808545.1	Contig	4.93753	57.8	19	4569
79	vB_OspX_pp79	31779	42	0	*Ochrobactrum* sp. 695/2009	695/2009	NZ_PCQT01000002.1	659993	691771	GCA_002808545.1	Contig	4.93753	57.8	19	4569
80	vB_OspX_pp80	24773	33	0	*Ochrobactrum* sp. 695/2009	695/2009	NZ_PCQT01000002.1	1623886	1648658	GCA_002808545.1	Contig	4.93753	57.8	19	4569
81	vB_OspX_pp81	32020	46	0	*Ochrobactrum* sp. 695/2009	695/2009	NZ_PCQT01000006.1	108922	140941	GCA_002808545.1	Contig	4.93753	57.8	19	4569
82	vB_OspX_pp82	21684	29	0	*Ochrobactrum* sp. 689/2009	689/2009	NZ_PCQU01000001.1	562472	584155	GCA_002808565.1	Contig	4.93586	57.8	13	4566
83	vB_OspX_pp83	15430	20	0	*Ochrobactrum* sp. 689/2009	689/2009	NZ_PCQU01000001.1	1615984	1631413	GCA_002808565.1	Contig	4.93586	57.8	13	4566
84	vB_OspX_pp84	40986	60	0	*Ochrobactrum* sp. 689/2009	689/2009	NZ_PCQU01000001.1	1802143	1843128	GCA_002808565.1	Contig	4.93586	57.8	13	4566
85	vB_OspX_pp85	56119	81	0	*Ochrobactrum* sp. 689/2009	689/2009	NZ_PCQU01000002.1	1	56119	GCA_002808565.1	Contig	4.93586	57.8	13	4566
86	vB_OspX_pp86	98726	127	0	*Ochrobactrum* sp. 30A/1000/2015	30A/1000/2015	NZ_PCFK01000002.1	392201	490926	GCA_002808585.1	Contig	4.86038	57.8	24	4568
87	vB_OspX_pp87	43636	62	0	*Ochrobactrum* sp. 30A/1000/2015	30A/1000/2015	NZ_PCFK01000004.1	102746	146381	GCA_002808585.1	Contig	4.86038	57.8	24	4568
88	vB_OspX_pp88	21459	28	0	*Ochrobactrum* sp. 30A/1000/2015	30A/1000/2015	NZ_PCFK01000005.1	47779	69237	GCA_002808585.1	Contig	4.86038	57.8	24	4568
89	vB_OspX_pp89	31363	35	0	*Ochrobactrum* sp. 30A/1000/2015	30A/1000/2015	NZ_PCFK01000011.1	301451	332813	GCA_002808585.1	Contig	4.86038	57.8	24	4568
90	vB_OspX_pp90	18597	33	0	*Ochrobactrum* sp. 30A/1000/2015	30A/1000/2015	NZ_PCFK01000015.1	1	18597	GCA_002808585.1	Contig	4.86038	57.8	24	4568
91	vB_OspX_pp91	42746	49	0	*Ochrobactrum* sp. 27A/999/2015	27A/999/2015	NZ_PCFL01000001.1	290011	332756	GCA_002808595.1	Contig	4.85653	57.8	23	4565

92	vB_OspX_pp92	11013	21	0	*Ochrobactrum* sp. 27A/999/2015	27A/999/2015	NZ_PCFL01000001.1	355202	366214	GCA_002808595.1	Contig	4.85653	57.8	23	4565
93	vB_OspX_pp93	22061	30	0	*Ochrobactrum* sp. 27A/999/2015	27A/999/2015	NZ_PCFL01000002.1	753410	775470	GCA_002808595.1	Contig	4.85653	57.8	23	4565
94	vB_OspX_pp94	44757	64	0	*Ochrobactrum* sp. 27A/999/2015	27A/999/2015	NZ_PCFL01000008.1	101864	146620	GCA_002808595.1	Contig	4.85653	57.8	23	4565
95	vB_OspX_pp95	18660	33	0	*Ochrobactrum* sp. 27A/999/2015	27A/999/2015	NZ_PCFL01000009.1	1	18660	GCA_002808595.1	Contig	4.85653	57.8	23	4565
96	vB_OspX_pp96	34582	39	0	*Ochrobactrum* sp. 27A/999/2015	27A/999/2015	NZ_PCFL01000011.1	97307	131888	GCA_002808595.1	Contig	4.85653	57.8	23	4565
97	vB_OspX_pp97	32619	36	0	*Ochrobactrum* sp. 23A/997/2015	23A/997/2015	NZ_PCFM01000001.1	1176298	1208916	GCA_002808625.1	Contig	4.85588	57.8	22	4566
98	vB_OspX_pp98	37034	48	0	*Ochrobactrum* sp. 23A/997/2015	23A/997/2015	NZ_PCFM01000002.1	752958	789991	GCA_002808625.1	Contig	4.85588	57.8	22	4566
99	vB_OspX_pp99	74388	97	0	*Ochrobactrum* sp. 23A/997/2015	23A/997/2015	NZ_PCFM01000005.1	446889	521276	GCA_002808625.1	Contig	4.85588	57.8	22	4566
100	vB_OspX_pp100	18715	33	0	*Ochrobactrum* sp. 23A/997/2015	23A/997/2015	NZ_PCFM01000011.1	43814	62528	GCA_002808625.1	Contig	4.85588	57.8	22	4566
101	vB_OspX_pp101	28623	46	0	*O. oryzae*	OA447	NZ_PTRC01000033.1	164	28786	GCA_002943495.1	Contig	4.46701	56.2	289	4108
102	vB_OspX_pp102	23420	41	0	*O. oryzae*	OA447	NZ_PTRC01000051.1	1	23420	GCA_002943495.1	Contig	4.46701	56.2	289	4108
103	vB_OspX_pp103	32234	41	0	*Ochrobactrum* sp. MYb19	MYb19	NZ_PCOM01000001.1	732255	764488	GCA_002975195.1	Scaffold	4.63173	53.4	21	4203
104	vB_OspX_pp104	26974	33	0	*Ochrobactrum* sp. MYb19	MYb19	NZ_PCOM01000002.1	737604	764577	GCA_002975195.1	Scaffold	4.63173	53.4	21	4203
105	vB_OspX_pp105	42587	61	1	*Ochrobactrum* sp. MYb71	MYb71	NZ_PCOC01000001.1	1219254	1261840	GCA_002975205.1	Contig	5.39977	55.9	3	4937
106	vB_OspX_pp106	35285	46	1	*Ochrobactrum* sp. MYb71	MYb71	NZ_PCOC01000001.1	1279395	1314679	GCA_002975205.1	Contig	5.39977	55.9	3	4937
107	vB_OspX_pp107	39241	54	2	*Ochrobactrum* sp. MYb71	MYb71	NZ_PCOC01000001.1	2609288	2648528	GCA_002975205.1	Contig	5.39977	55.9	3	4937
108	vB_OspX_pp108	15631	20	0	*O. pseudogrignonense*	MYb58	NZ_PCOG01000001.1	598914	614544	GCA_002975235.1	Contig	4.97449	53.6	3	4476
109	vB_OspX_pp109	30209	35	0	*O. pseudogrignonense*	MYb58	NZ_PCOG01000001.1	977330	1007538	GCA_002975235.1	Contig	4.97449	53.6	3	4476
110	vB_OspX_pp110	42405	52	0	*O. pseudogrignonense*	MYb58	NZ_PCOG01000001.1	1082670	1125074	GCA_002975235.1	Contig	4.97449	53.6	3	4476
111	vB_OspX_pp111	83481	111	3	*Ochrobactrum* sp. MYb49	MYb49	NZ_PCOI01000002.1	810276	893756	GCA_002975255.1	Contig	4.825	55.9	3	4413
112	vB_OspX_pp112	30427	36	0	*O. pseudogrignonense*	MYb70	NZ_PCOD01000001.1	243786	274212	GCA_002979215.1	Scaffold	4.93647	53.6	17	4463
113	vB_OspX_pp113	44340	54	0	*O. pseudogrignonense*	MYb70	NZ_PCOD01000001.1	348424	392763	GCA_002979215.1	Scaffold	4.93647	53.6	17	4463
114	vB_OspX_pp114	26045	25	0	*O. pseudogrignonense*	MYb70	NZ_PCOD01000004.1	66264	92308	GCA_002979215.1	Scaffold	4.93647	53.6	17	4463
115	vB_OspX_pp115	19711	22	0	*Ochrobactrum* sp. MYb68	MYb68	NZ_PCOE01000001.1	37857	57567	GCA_002979225.1	Contig	5.05836	53.3	41	4628
116	vB_OspX_pp116	42134	68	0	*Ochrobactrum* sp. MYb68	MYb68	NZ_PCOE01000005.1	1	42134	GCA_002979225.1	Contig	5.05836	53.3	41	4628
117	vB_OspX_pp117	43824	54	0	*O. pseudogrignonense*	MYb37	NZ_PCOK01000001.1	745312	789135	GCA_002979295.1	Scaffold	4.9392	53.6	19	4465
118	vB_OspX_pp118	31730	38	0	*O. pseudogrignonense*	MYb37	NZ_PCOK01000001.1	862350	894079	GCA_002979295.1	Scaffold	4.9392	53.6	19	4465
119	vB_OspX_pp119	24070	31	0	*Ochrobactrum* sp. MYb18	MYb18	NZ_PCON01000001.1	740441	764510	GCA_002979315.1	Contig	4.63235	53.4	23	4208

120	vB_OspX_pp120	38344	47	0	*Ochrobactrum* sp. MYb18	MYb18	NZ_PCON01000006.1	10736	49079	GCA_002979315.1	Contig	4.63235	53.4	23	4208
121	vB_OspX_pp121	37603	45	0	*O. thiophenivorans*	MYb6	NZ_PCOR01000001.1	1071202	1108804	GCA_002979335.1	Contig	4.65756	53.4	3	4208
122	vB_OspX_pp122	44253	55	0	*O. thiophenivorans*	MYb6	NZ_PCOR01000002.1	168666	212918	GCA_002979335.1	Contig	4.65756	53.4	3	4208
123	vB_OspX_pp123	31689	41	0	*Ochrobactrum* sp. MYb29	MYb29	NZ_PCOL01000002.1	300486	332174	GCA_002979345.1	Contig	4.8203	53.2	106	4349
124	vB_OspX_pp124	17423	21	0	*Ochrobactrum* sp. MYb29	MYb29	NZ_PCOL01000003.1	55	17477	GCA_002979345.1	Contig	4.8203	53.2	106	4349
125	vB_OspX_pp125	37316	44	0	*Ochrobactrum* sp. MYb15	MYb15	NZ_PCOO01000001.1	1071202	1108517	GCA_002979375.1	Contig	4.65756	53.4	3	4208
126	vB_OspX_pp126	44448	55	0	*Ochrobactrum* sp. MYb15	MYb15	NZ_PCOO01000002.1	790541	834988	GCA_002979375.1	Contig	4.65756	53.4	3	4208
127	vB_OspX_pp127	24605	23	2	*Ochrobactrum* sp. MYb14	MYb14	NZ_PCOP01000001.1	985854	1010458	GCA_002979395.1	Contig	4.63214	53.4	20	4206
128	vB_OspX_pp128	24760	31	0	*Ochrobactrum* sp. MYb14	MYb14	NZ_PCOP01000003.1	738955	763714	GCA_002979395.1	Contig	4.63214	53.4	20	4206
129	vB_OspX_pp129	34400	53	0	*O. pituitosum*	CCUG 50899	NZ_PYSY02000001.1	960235	994634	GCA_003049685.2	Contig	5.51819	53.4	10	4803
130	vB_OspX_pp130	69165	88	0	*O. pituitosum*	CCUG 50899	NZ_PYSY02000002.1	747578	816742	GCA_003049685.2	Contig	5.51819	53.4	10	4803
131	vB_OspX_pp131	43102	52	0	*O. pituitosum*	CCUG 50899	NZ_PYSY02000002.1	1458961	1502062	GCA_003049685.2	Contig	5.51819	53.4	10	4803
132	vB_OspX_pp132	57435	78	2	*Ochrobactrum* sp. POC9	POC9	NZ_QGST01000002.1	15085	72519	GCA_003176975.1	Contig	4.96957	55.7	255	4617
133	vB_OspX_pp133	9866	13	0	*Ochrobactrum* sp. POC9	POC9	NZ_QGST01000010.1	65629	75494	GCA_003176975.1	Contig	4.96957	55.7	255	4617
134	vB_OspX_pp134	38480	47	1	*Ochrobactrum* sp. POC9	POC9	NZ_QGST01000013.1	16703	55182	GCA_003176975.1	Contig	4.96957	55.7	255	4617
135	vB_OspX_pp135	14206	14	0	*Ochrobactrum* sp. POC9	POC9	NZ_QGST01000014.1	95231	109436	GCA_003176975.1	Contig	4.96957	55.7	255	4617
136	vB_OspX_pp136	29118	39	0	*Ochrobactrum* sp. POC9	POC9	NZ_QGST01000019.1	17264	46381	GCA_003176975.1	Contig	4.96957	55.7	255	4617
137	vB_OspX_pp137	46092	57	0	*Ochrobactrum* sp. 3-3	3-3	NZ_LWHH01000001.1	418257	464348	GCA_003332115.1	Contig	4.95132	57.3	36	4634
138	vB_OspX_pp138	36995	48	1	*Ochrobactrum* sp. 3-3	3-3	NZ_LWHH01000004.1	373235	410229	GCA_003332115.1	Contig	4.95132	57.3	36	4634
139	vB_OspX_pp139	30539	48	0	*Ochrobactrum* sp. 3-3	3-3	NZ_LWHH01000010.1	109759	140297	GCA_003332115.1	Contig	4.95132	57.3	36	4634
140	vB_OspX_pp140	11846	19	0	*O. anthropi*	UBA11634	DOGY01000370.1	157	12002	GCA_003528245.1	Scaffold	3.92964	55.4	646	4119
141	vB_OspX_pp141	44396	51	0	*O. haematophilum*	L1Suc	NZ_QXCX01000002.1	1391229	1435624	GCA_003550135.1	Contig	4.91016	59.1	3	4418
142	vB_OspX_pp142	33639	47	1	*O. haematophilum*	L1Suc	NZ_QXCX01000003.1	130747	164385	GCA_003550135.1	Contig	4.91016	59.1	3	4418
143	vB_OspX_pp143	51020	73	2	*Ochrobactrum* sp. BO-7	BO-7	NZ_RCHH01000006.1	1	51020	GCA_003664555.1	Contig	5.02412	57.2	48	4568
144	vB_OspX_pp144	32191	43	1	*Ochrobactrum* sp. DDT2	DDT2	NZ_LKAD01000006.1	33578	65768	GCA_003702725.1	Scaffold	4.63036	56	24	4275
145	vB_OspX_pp145	40626	57	1	*Ochrobactrum* sp. DDT2	DDT2	NZ_LKAD01000013.1	239094	279719	GCA_003702725.1	Scaffold	4.63036	56	24	4275
146	vB_OspX_pp146	42668	47	0	*O. anthropi*	OA_392	NZ_RHTC01000001.1	638918	681585	GCA_003937425.1	Scaffold	5.06671	56.4	41	4789
147	vB_OspX_pp147	32681	43	0	*O. anthropi*	OA_392	NZ_RHTC01000002.1	331326	364006	GCA_003937425.1	Scaffold	5.06671	56.4	41	4789
148	vB_OspX_pp148	64507	96	0	*O. anthropi*	OA_392	NZ_RHTC01000004.1	212721	277227	GCA_003937425.1	Scaffold	5.06671	56.4	41	4789
149	vB_OspX_pp149	27720	40	0	*O. anthropi*	OA_392	NZ_RHTC01000004.1	430108	457827	GCA_003937425.1	Scaffold	5.06671	56.4	41	4789
150	vB_OspX_pp150	11436	21	1	*O. anthropi*	OA_392	NZ_RHTC01000011.1	1	11436	GCA_003937425.1	Scaffold	5.06671	56.4	41	4789
151	vB_OspX_pp151	24292	34	0	*O. anthropi*	OA_392	NZ_RHTC01000014.1	3474	27765	GCA_003937425.1	Scaffold	5.06671	56.4	41	4789
152	vB_OspX_pp152	48390	73	1	*O. anthropi*	OA_244	NZ_RHTD01000001.1	881924	930313	GCA_003937445.1	Scaffold	5.02056	56.1	79	4692
153	vB_OspX_pp153	37616	51	0	*O. anthropi*	OA_244	NZ_RHTD01000010.1	45014	82629	GCA_003937445.1	Scaffold	5.02056	56.1	79	4692

154	vB_OspX_pp154	32782	38	0	*O. anthropi*	OA_244	NZ_RHTD01000010.1	129032	161813	GCA_003937445.1	Scaffold	5.02056	56.1	79	4692
155	vB_OspX_pp155	16015	22	0	*O. anthropi*	OA_244	NZ_RHTD01000012.1	123645	139659	GCA_003937445.1	Scaffold	5.02056	56.1	79	4692
156	vB_OspX_pp156	36630	49	0	*Ochrobactrum* sp. AV	AV	NZ_SADZ01000002.1	113889	150518	GCA_004011925.1	Scaffold	4.94999	58.2	1119	4458
157	vB_OspX_pp157	59755	78	2	*Ochrobactrum* sp. AV	AV	NZ_SADZ01000002.1	479496	539250	GCA_004011925.1	Scaffold	4.94999	58.2	1119	4458
158	vB_OspX_pp158	23367	22	0	*Ochrobactrum* sp. BH3	BH3	NZ_SLYR01000001.1	1007081	1030447	GCA_004341145.1	Contig	4.36519	53.3	48	3986
159	vB_OspX_pp159	29772	39	2	*Ochrobactrum* sp. BH3	BH3	NZ_SLYR01000003.1	161	29932	GCA_004341145.1	Contig	4.36519	53.3	48	3986
160	vB_OspX_pp160	17157	26	0	*Ochrobactrum* sp. BH3	BH3	NZ_SLYR01000003.1	297154	314310	GCA_004341145.1	Contig	4.36519	53.3	48	3986
161	vB_OspX_pp161	21356	26	0	*Ochrobactrum* sp. BH3	BH3	NZ_SLYR01000008.1	99382	120737	GCA_004341145.1	Contig	4.36519	53.3	48	3986
162	vB_OspX_pp162	46818	65	0	*Ochrobactrum* sp. LP_5_YM	LP_5_YM	NZ_SOCS01000001.1	852656	899473	GCA_004368705.1	Scaffold	5.05243	52.6	47	4580
163	vB_OspX_pp163	23843	32	0	*Ochrobactrum* sp. LP_5_YM	LP_5_YM	NZ_SOCS01000002.1	555520	579362	GCA_004368705.1	Scaffold	5.05243	52.6	47	4580
164	vB_OspX_pp164	52948	77	0	*Ochrobactrum* sp. LP_5_YM	LP_5_YM	NZ_SOCS01000004.1	151728	204675	GCA_004368705.1	Scaffold	5.05243	52.6	47	4580
165	vB_OspX_pp165	18780	20	0	*Ochrobactrum* sp. LP_5_YM	LP_5_YM	NZ_SOCS01000005.1	13358	32137	GCA_004368705.1	Scaffold	5.05243	52.6	47	4580
166	vB_OspX_pp166	38979	51	2	*Ochrobactrum* sp. CPD-03	CPD-03	NZ_RSEU01000002.1	788245	827223	GCA_005930505.1	Scaffold	4.66006	57.7	11	4321
167	vB_OspX_pp167	30925	39	0	*Ochrobactrum* sp. CPD-03	CPD-03	NZ_RSEU01000003.1	2258035	2288959	GCA_005930505.1	Scaffold	4.66006	57.7	11	4321
168	vB_OspX_pp168	35461	42	0	*O. haematophilum*	CCUG 38531	NZ_VCPE01000001.1	106253	141713	GCA_005938105.1	Contig	5.50326	56.7	55	5013
169	vB_OspX_pp169	28732	40	0	*O. haematophilum*	CCUG 38531	NZ_VCPE01000001.1	441297	470028	GCA_005938105.1	Contig	5.50326	56.7	55	5013
170	vB_OspX_pp170	66990	92	1	*O. haematophilum*	CCUG 38531	NZ_VCPE01000006.1	143039	210028	GCA_005938105.1	Contig	5.50326	56.7	55	5013
171	vB_OspX_pp171	18198	22	0	*Ochrobactrum* sp. CGA5	CGA5	NZ_VCIG01000038.1	43426	61623	GCA_006345815.1	Scaffold	4.60274	55.2	51	4238
172	vB_OspX_pp172	44991	72	1	*Ochrobactrum* sp. CGA5	CGA5	NZ_VCIG01000038.1	438142	483132	GCA_006345815.1	Scaffold	4.60274	55.2	51	4238
173	vB_OspX_pp173	52275	67	0	*O. pecoris*	08RB2639	NZ_VEWK01000002.1	2131	54405	GCA_006376675.1	Contig	5.05734	55.9	61	4715
174	vB_OspX_pp174	35076	46	1	*O. pecoris*	08RB2639	NZ_VEWK01000002.1	191777	226852	GCA_006376675.1	Contig	5.05734	55.9	61	4715
175	vB_OspX_pp175	16931	30	0	*O. pecoris*	08RB2639	NZ_VEWK01000013.1	100050	116980	GCA_006376675.1	Contig	5.05734	55.9	61	4715
176	vB_OspX_pp176	32007	45	0	*Ochrobactrum* sp. LCB8	LCB8	NZ_VEWL01000002.1	397911	429917	GCA_006376685.1	Contig	4.7608	57.1	43	4288
177	vB_OspX_pp177	18311	30	1	*Ochrobactrum* sp. LCB8	LCB8	NZ_VEWL01000010.1	130294	148604	GCA_006376685.1	Contig	4.7608	57.1	43	4288
178	vB_OspX_pp178	43808	62	0	*O. gallinifaecis*	ISO 196	NZ_ML636801.1	415252	459059	GCA_006476605.1	Scaffold	3.74242	51	37	3366
179	vB_OspX_pp179	20678	25	0	*O. gallinifaecis*	ISO 196	NZ_ML636805.1	127655	148332	GCA_006476605.1	Scaffold	3.74242	51	37	3366
180	vB_OspX_pp180	15689	28	0	*Ochrobactrum* sp. J50	J50	NZ_VLJR01000005.1	114309	129997	GCA_007829595.1	Scaffold	4.2154	57.7	60	3975
181	vB_OspX_pp181	32762	42	0	*O. quorumnocens*	RPTAtOch1	NZ_VYXQ01000004.1	12295	45056	GCA_008728025.1	Contig	5.05264	53.5	77	4539
182	vB_OspX_pp182	31573	50	0	*O. pituitosum*	CCUG 50899	NZ_VZPE01000001.1	505695	537267	GCA_008801705.1	Contig	5.17044	53.5	60	4759
183	vB_OspX_pp183	43775	53	0	*O. pituitosum*	CCUG 50899	NZ_VZPE01000004.1	104030	147804	GCA_008801705.1	Contig	5.17044	53.5	60	4759
184	vB_OspX_pp184	64359	86	0	*O. pituitosum*	CCUG 50899	NZ_VZPE01000006.1	169173	233531	GCA_008801705.1	Contig	5.17044	53.5	60	4759
185	vB_OspX_pp185	22741	26	0	*O. tritici*	LMG 401	NZ_WBVZ01000016.1	22211	44951	GCA_008932275.1	Contig	4.96805	56	141	4640
186	vB_OspX_pp186	48005	74	1	*O. tritici*	TA93	NZ_WBVY01000004.1	179178	227182	GCA_008932285.1	Contig	5.02572	55.8	44	4689
187	vB_OspX_pp187	30514	38	0	*O. tritici*	TA93	NZ_WBVY01000004.1	603331	633844	GCA_008932285.1	Contig	5.02572	55.8	44	4689

188	vB_OspX_pp188	15651	21	0	*O. tritici*	TA93	NZ_WBVY01000008.1	47895	63545	GCA_008932285.1	Contig	5.02572	55.8	44	4689
189	vB_OspX_pp189	54437	75	1	*O. tritici*	LMG 18957	NZ_WBWA01000002.1	183	54619	GCA_008932295.1	Contig	5.203	55.9	153	4921
190	vB_OspX_pp190	39014	53	0	*O. tritici*	LMG 18957	NZ_WBWA01000003.1	147599	186612	GCA_008932295.1	Contig	5.203	55.9	153	4921
191	vB_OspX_pp191	64591	90	0	*O. tritici*	LMG 18957	NZ_WBWA01000005.1	143024	207614	GCA_008932295.1	Contig	5.203	55.9	153	4921
192	vB_OspX_pp192	22645	32	1	*O. tritici*	LMG 18957	NZ_WBWA01000008.1	38785	61429	GCA_008932295.1	Contig	5.203	55.9	153	4921
193	vB_OspX_pp193	22178	20	0	*O. tritici*	LMG 18957	NZ_WBWA01000028.1	363	22540	GCA_008932295.1	Contig	5.203	55.9	153	4921
194	vB_OspX_pp194	55722	75	0	*O. tritici*	WS1830	NZ_WBVX01000002.1	116065	171786	GCA_008932305.1	Contig	5.88771	56.3	74	5577
195	vB_OspX_pp195	36588	49	0	*O. tritici*	WS1830	NZ_WBVX01000006.1	172955	209542	GCA_008932305.1	Contig	5.88771	56.3	74	5577
196	vB_OspX_pp196	34189	36	0	*O. tritici*	WS1830	NZ_WBVX01000016.1	120	34308	GCA_008932305.1	Contig	5.88771	56.3	74	5577
197	vB_OspX_pp197	22088	23	0	*O. tritici*	WS1830	NZ_WBVX01000028.1	272	22359	GCA_008932305.1	Contig	5.88771	56.3	74	5577
198	vB_OspX_pp198	31051	40	1	*O. tritici*	WS1830	NZ_WBVX01000030.1	62	31112	GCA_008932305.1	Contig	5.88771	56.3	74	5577
199	vB_OspX_pp199	36437	44	2	*Ochrobactrum* sp. Kaboul	Kaboul	NZ_WBWC01000002.1	415178	451614	GCA_008932375.1	Scaffold	4.69577	57.2	56	4355
200	vB_OspX_pp200	77000	102	1	*Ochrobactrum* sp. Kaboul	Kaboul	NZ_WBWC01000004.1	269130	346129	GCA_008932375.1	Scaffold	4.69577	57.2	56	4355
201	vB_OspX_pp201	25118	39	0	*O. lupini*	LUP23	NZ_WBWF01000027.1	38515	63632	GCA_008932385.1	Contig	5.43096	56.4	100	5057
202	vB_OspX_pp202	22812	32	1	*Ochrobactrum* sp. LMG 5442	LMG 5442	NZ_WBWB01000001.1	250561	273372	GCA_008932395.1	Contig	4.61154	57.7	15	4297
203	vB_OspX_pp203	23382	35	0	*Ochrobactrum* sp. LMG 5442	LMG 5442	NZ_WBWB01000002.1	1366549	1389930	GCA_008932395.1	Contig	4.61154	57.7	15	4297
204	vB_OspX_pp204	51764	72	1	*Ochrobactrum* sp. LMG 5442	LMG 5442	NZ_WBWB01000003.1	212374	264137	GCA_008932395.1	Contig	4.61154	57.7	15	4297
205	vB_OspX_pp205	34111	44	0	*O. pseudogrignonense*	CCUG 43892	NZ_WBWD01000001.1	1809627	1843737	GCA_008932425.1	Contig	4.97946	53.9	35	4548
206	vB_OspX_pp206	37188	41	0	*O. pseudogrignonense*	CCUG 43892	NZ_WBWD01000003.1	326934	364121	GCA_008932425.1	Contig	4.97946	53.9	35	4548
207	vB_OspX_pp207	31754	39	0	*O. pseudintermedium*	CCUG 34735	NZ_WBWE01000002.1	319292	351045	GCA_008932435.1	Contig	4.39464	57.9	45	4168
208	vB_OspX_pp208	54271	73	0	*O. pseudintermedium*	CCUG 34735	NZ_WBWE01000005.1	98157	152427	GCA_008932435.1	Contig	4.39464	57.9	45	4168
209	vB_OspX_pp209	48147	74	1	*O. pseudintermedium*	CCUG 34735	NZ_WBWE01000013.1	1	48147	GCA_008932435.1	Contig	4.39464	57.9	45	4168
210	vB_OspX_pp210	35270	41	0	*O. intermedium*	TM73	NZ_WBWG01000005.1	147726	182995	GCA_008932475.1	Contig	4.44412	57.9	67	4152
211	vB_OspX_pp211	15627	29	1	*O. intermedium*	TM73	NZ_WBWG01000007.1	175934	191560	GCA_008932475.1	Contig	4.44412	57.9	67	4152
212	vB_OspX_pp212	62316	92	1	*O. intermedium*	OiC8-6	NZ_WBWI01000004.1	88574	150889	GCA_008932485.1	Contig	4.85104	57.8	60	4520
213	vB_OspX_pp213	20962	36	2	*O. intermedium*	OiC8-6	NZ_WBWI01000006.1	38937	59898	GCA_008932485.1	Contig	4.85104	57.8	60	4520
214	vB_OspX_pp214	65965	95	1	*O. intermedium*	TD30	NZ_WBWH01000003.1	91103	157067	GCA_008932495.1	Contig	4.53155	57.8	36	4242
215	vB_OspX_pp215	19257	30	1	*O. intermedium*	TD30	NZ_WBWH01000003.1	447814	467070	GCA_008932495.1	Contig	4.53155	57.8	36	4242
216	vB_OspX_pp216	9084	20	0	*O. intermedium*	TD30	NZ_WBWH01000006.1	69610	78693	GCA_008932495.1	Contig	4.53155	57.8	36	4242
217	vB_OspX_pp217	62523	100	2	*O. intermedium*	LMG 379	NZ_WBWJ01000002.1	346532	409054	GCA_008932505.1	Contig	4.64469	57.7	99	4354
218	vB_OspX_pp218	45835	60	0	*O. intermedium*	CCM 7036	NZ_WBWL01000001.1	212061	257895	GCA_008932575.1	Contig	4.84627	57.6	83	4476
219	vB_OspX_pp219	35346	46	1	*O. intermedium*	CCM 7036	NZ_WBWL01000004.1	407386	442731	GCA_008932575.1	Contig	4.84627	57.6	83	4476
220	vB_OspX_pp220	23106	30	2	*O. intermedium*	CCM 7036	NZ_WBWL01000007.1	266	23371	GCA_008932575.1	Contig	4.84627	57.6	83	4476
221	vB_OspX_pp221	30505	47	0	*O. intermedium*	CCM 7036	NZ_WBWL01000014.1	41031	71535	GCA_008932575.1	Contig	4.84627	57.6	83	4476

222	vB_OspX_pp222	47350	72	1	*O. anthropi*	LMG 7991	NZ_WBWM01000001.1	360858	408207	GCA_008932585.1	Contig	4.80673	56.1	12	4432
223	vB_OspX_pp223	25302	28	0	*O. anthropi*	LMG 7991	NZ_WBWM01000005.1	225007	250308	GCA_008932585.1	Contig	4.80673	56.1	12	4432
224	vB_OspX_pp224	42099	57	2	*O. anthropi*	LMG 5140	NZ_WBWN01000003.1	160506	202604	GCA_008932615.1	Contig	4.97834	56.2	70	4604
225	vB_OspX_pp225	46142	67	1	*O. anthropi*	LMG 34	NZ_WBWP01000001.1	368982	415123	GCA_008932625.1	Contig	4.80815	56.1	10	4431
226	vB_OspX_pp226	25817	29	0	*O. anthropi*	LMG 34	NZ_WBWP01000006.1	224492	250308	GCA_008932625.1	Contig	4.80815	56.1	10	4431
227	vB_OspX_pp227	33573	41	0	*O. anthropi*	LMG 371	NZ_WBWO01000001.1	254527	288099	GCA_008932635.1	Contig	4.85461	56.3	20	4518
228	vB_OspX_pp228	99638	133	0	*O. anthropi*	LMG 371	NZ_WBWO01000001.1	768509	868146	GCA_008932635.1	Contig	4.85461	56.3	20	4518
229	vB_OspX_pp229	18468	24	0	*O. anthropi*	LMG 371	NZ_WBWO01000004.1	341133	359600	GCA_008932635.1	Contig	4.85461	56.3	20	4518
230	vB_OspX_pp230	48987	52	0	*O. anthropi*	LMG 3333	NZ_WBWQ01000001.1	127678	176664	GCA_008932675.1	Contig	5.0078	56.4	23	4644
231	vB_OspX_pp231	25723	32	0	*O. anthropi*	LMG 3333	NZ_WBWQ01000011.1	6289	32011	GCA_008932675.1	Contig	5.0078	56.4	23	4644
232	vB_OspX_pp232	36249	49	1	*O. anthropi*	LMG 3329	NZ_WBWR01000001.1	225861	262109	GCA_008932685.1	Contig	5.19137	56.3	76	4853
233	vB_OspX_pp233	42607	58	1	*O. anthropi*	LMG 3329	NZ_WBWR01000003.1	107789	150395	GCA_008932685.1	Contig	5.19137	56.3	76	4853
234	vB_OspX_pp234	41550	65	1	*O. anthropi*	LMG 3329	NZ_WBWR01000003.1	650736	692285	GCA_008932685.1	Contig	5.19137	56.3	76	4853
235	vB_OspX_pp235	16340	30	0	*O. anthropi*	LMG 3313	NZ_WBWS01000005.1	195838	212177	GCA_008932705.1	Contig	5.31386	56.5	161	4991
236	vB_OspX_pp236	33777	47	0	*O. anthropi*	LMG 3313	NZ_WBWS01000006.1	109520	143296	GCA_008932705.1	Contig	5.31386	56.5	161	4991
237	vB_OspX_pp237	49873	67	1	*O. anthropi*	LMG 3313	NZ_WBWS01000012.1	125233	175105	GCA_008932705.1	Contig	5.31386	56.5	161	4991
238	vB_OspX_pp238	28922	39	0	*O. anthropi*	LMG 3313	NZ_WBWS01000016.1	58299	87220	GCA_008932705.1	Contig	5.31386	56.5	161	4991
239	vB_OspX_pp239	40473	51	0	*O. anthropi*	LMG 3307	NZ_WBWT01000003.1	307511	347983	GCA_008932725.1	Contig	4.91285	56.1	41	4556
240	vB_OspX_pp240	37576	50	0	*O. anthropi*	LMG 3307	NZ_WBWT01000006.1	176336	213911	GCA_008932725.1	Contig	4.91285	56.1	41	4556
241	vB_OspX_pp241	37025	48	1	*O. anthropi*	LMG 3298	NZ_WBWU01000001.1	783996	821020	GCA_008932745.1	Contig	4.9583	56.2	33	4630
242	vB_OspX_pp242	30006	41	1	*O. anthropi*	LMG 3298	NZ_WBWU01000002.1	566497	596502	GCA_008932745.1	Contig	4.9583	56.2	33	4630
243	vB_OspX_pp243	41153	49	0	*O. anthropi*	LMG 3298	NZ_WBWU01000003.1	561433	602585	GCA_008932745.1	Contig	4.9583	56.2	33	4630
244	vB_OspX_pp244	38914	49	1	*O. anthropi*	LMG 2136	NZ_WBWV01000003.1	474817	513730	GCA_008932765.1	Contig	4.90489	56.4	37	4511
245	vB_OspX_pp245	37679	47	1	*O. anthropi*	DSM 14396	NZ_WBWW01000003.1	256445	294123	GCA_008932785.1	Contig	4.79777	56	31	4398
246	vB_OspX_pp246	37627	42	0	*O. anthropi*	CCUG 34461	NZ_WBWX01000001.1	731642	769268	GCA_008932815.1	Contig	5.44654	56.5	51	5061
247	vB_OspX_pp247	49945	60	0	*O. anthropi*	CCUG 34461	NZ_WBWX01000002.1	557637	607581	GCA_008932815.1	Contig	5.44654	56.5	51	5061
248	vB_OspX_pp248	48433	67	0	*O. anthropi*	CCUG 34461	NZ_WBWX01000003.1	220321	268753	GCA_008932815.1	Contig	5.44654	56.5	51	5061
249	vB_OspX_pp249	37520	42	0	*O. anthropi*	CCUG 34461	NZ_WBWX01000011.1	64034	101553	GCA_008932815.1	Contig	5.44654	56.5	51	5061
250	vB_OspX_pp250	44174	70	0	*O. anthropi*	CCUG 25934	NZ_WBWZ01000002.1	204154	248327	GCA_008932825.1	Contig	5.14201	56.1	27	4799
251	vB_OspX_pp251	63686	86	1	*O. anthropi*	CCUG 25934	NZ_WBWZ01000002.1	516702	580387	GCA_008932825.1	Contig	5.14201	56.1	27	4799
252	vB_OspX_pp252	34688	45	1	*O. anthropi*	CCUG 33786	NZ_WBWY01000001.1	751125	785812	GCA_008932835.1	Contig	5.179	56.4	65	4855
253	vB_OspX_pp253	22055	31	1	*O. anthropi*	CCUG 33786	NZ_WBWY01000002.1	616610	638664	GCA_008932835.1	Contig	5.179	56.4	65	4855
254	vB_OspX_pp254	57359	79	1	*O. anthropi*	CCUG 33786	NZ_WBWY01000005.1	146170	203528	GCA_008932835.1	Contig	5.179	56.4	65	4855
255	vB_OspX_pp255	40719	46	0	*O. anthropi*	CCUG 33786	NZ_WBWY01000011.1	20374	61092	GCA_008932835.1	Contig	5.179	56.4	65	4855

256	vB_OspX_pp256	26968	31	0	*O. anthropi*	CCUG 33786	NZ_WBWY01000012.1	51988	78955	GCA_008932835.1	Contig	5.179	56.4	65	4855
257	vB_OspX_pp257	20192	26	0	*O. anthropi*	CCUG 33786	NZ_WBWY01000014.1	68080	88271	GCA_008932835.1	Contig	5.179	56.4	65	4855
258	vB_OspX_pp258	26553	37	1	*O. anthropi*	CCUG 12415	NZ_WBXA01000001.1	436858	463410	GCA_008932875.1	Contig	5.11931	56.4	72	4773
259	vB_OspX_pp259	37812	50	0	*O. anthropi*	CCUG 12415	NZ_WBXA01000002.1	27002	64813	GCA_008932875.1	Contig	5.11931	56.4	72	4773
260	vB_OspX_pp260	26185	33	0	*O. anthropi*	CCUG 12415	NZ_WBXA01000003.1	334797	360981	GCA_008932875.1	Contig	5.11931	56.4	72	4773
261	vB_OspX_pp261	14872	21	0	*O. anthropi*	CCM 4352	NZ_WBXB01000001.1	1248996	1263867	GCA_008932885.1	Contig	4.83915	56.1	21	4442
262	vB_OspX_pp262	45854	67	1	*O. anthropi*	ALM4	NZ_WBXC01000001.1	360858	406711	GCA_008932905.1	Contig	4.81384	56.1	17	4435
263	vB_OspX_pp263	25817	29	0	*O. anthropi*	ALM4	NZ_WBXC01000005.1	224492	250308	GCA_008932905.1	Contig	4.81384	56.1	17	4435
264	vB_OspX_pp264	40174	52	1	*O. anthropi*	T16R-87	CP044970.1	306130	346303	GCA_009017375.1	Complete	4.73688	55.991	2	4324
265	vB_OspX_pp265	46599	57	1	*O. anthropi*	T16R-87	CP044971.1	1764484	1811082	GCA_009017375.1	Complete	4.73688	55.991	2	4324
266	vB_OspX_pp266	59213	82	1	*O. haematophilum*	FI11154	NZ_OOFM01000004.1	318251	377463	GCA_900243435.1	Contig	5.4823	57	5	4965
267	vB_OspX_pp267	22946	29	1	*O. haematophilum*	FI11154	NZ_OOFM01000004.1	665728	688673	GCA_900243435.1	Contig	5.4823	57	5	4965
268	vB_OspX_pp268	32548	40	1	*O. haematophilum*	FI11154	NZ_OOFM01000004.1	822656	855203	GCA_900243435.1	Contig	5.4823	57	5	4965
269	vB_OspX_pp269	30050	37	0	*O. haematophilum*	FI11154	NZ_OOFM01000004.1	958244	988293	GCA_900243435.1	Contig	5.4823	57	5	4965
270	vB_OspX_pp270	30494	40	0	*O. haematophilum*	FI11154	NZ_OOFM01000005.1	285626	316119	GCA_900243435.1	Contig	5.4823	57	5	4965
271	vB_OspX_pp271	67644	93	1	*O. haematophilum*	FI11154	NZ_OOFM01000005.1	1180872	1248515	GCA_900243435.1	Contig	5.4823	57	5	4965
272	vB_OspX_pp272	40711	45	0	*O. intermedium*	NCTC12171	NZ_UGSH01000001.1	19	40729	GCA_900454225.1	Contig	4.72789	57.7	3	4348
273	vB_OspX_pp273	41665	52	1	*O. intermedium*	NCTC12171	NZ_UGSH01000002.1	7936	49600	GCA_900454225.1	Contig	4.72789	57.7	3	4348
274	vB_OspX_pp274	35856	48	0	*O. intermedium*	NCTC12171	NZ_UGSH01000003.1	1799801	1835656	GCA_900454225.1	Contig	4.72789	57.7	3	4348
275	vB_OspX_pp275	33082	57	0	*O. anthropi*	NCTC12168	NZ_UGSA01000001.1	223209	256290	GCA_900454235.1	Contig	5.23976	56.1	5	4774
276	vB_OspX_pp276	35997	46	1	*O. anthropi*	NCTC12168	NZ_UGSA01000001.1	1178329	1214325	GCA_900454235.1	Contig	5.23976	56.1	5	4774
277	vB_OspX_pp277	63204	97	0	*O. anthropi*	NCTC12168	NZ_UGSA01000001.1	1990552	2053755	GCA_900454235.1	Contig	5.23976	56.1	5	4774
Ochrobactrum spp. in which prophage regions were not identified															
-	-	-	-	-	*O. anthropi*	SUBG007	-	-	-	GCA_001586735.1	Contig	4.37595	54	8113	1642
-	-	-	-	-	*Ochrobactrum* sp.	UBA8664	-	-	-	GCA_003523145.1	Scaffold	3.44006	57	576	2584
-	-	-	-	-	*O. intermedium*	BP8.5	-	-	-	GCA_003852825.1	Contig	2.91651	58.4	1146	3162
-	-	-	-	-	*O. anthropi* 60a	60a	-	-	-	GCA_000409625.1	Contig	4.59002	56.1	1004	4062
-	-	-	-	-	*O. intermedium*	CCUG 39736	-	-	-	GCA_008932555.1	Contig	4.47937	58.2	26	4105
-	-	-	-	-	*Ochrobactrum* sp. MH181795	MH181795	-	-	-	GCA_003725855.1	Contig	4.56429	56	15	4156
-	-	-	-	-	*O. intermedium* M86	M86	-	-	-	GCA_001996385.1	Contig	4.68176	57.7	121	4297
-	-	-	-	-	*O. anthropi*	DE2010	-	-	-	GCA_003325675.1	Contig	4.90469	56.5	26	4478
Ochrobactrum spp. not included in prophage search due to various technical reasons															

-	-	-	-	-	*O. anthropi*	UBA3888	-	-	-	GCA_002391935.1	Scaffold	4.12451	55.5	294	0
-	-	-	-	-	*O. anthropi*	UBA4550	-	-	-	GCA_002387885.1	Scaffold	4.91087	55.9	50	0
-	-	-	-	-	*O. anthropi*	UBA5925	-	-	-	GCA_002431065.1	Scaffold	4.803	56.1	33	0
-	-	-	-	-	*O. anthropi*	UBA6689	-	-	-	GCA_002454835.1	Scaffold	4.59989	56.1	28	0
-	-	-	-	-	*O. anthropi*	UBA6743	-	-	-	GCA_002462635.1	Scaffold	5.07442	56	36	0
-	-	-	-	-	*O. anthropi*	UBA6763	-	-	-	GCA_002453395.1	Scaffold	3.81333	55.7	331	0
-	-	-	-	-	*O. anthropi*	UBA7245	-	-	-	GCA_002472885.1	Scaffold	4.79606	55.9	64	0
-	-	-	-	-	*O. anthropi*	UBA7489	-	-	-	GCA_002477165.1	Scaffold	4.5845	56	26	0
-	-	-	-	-	*O. anthropi*	UBA809	-	-	-	GCA_002296345.1	Scaffold	4.97784	56	35	0
-	-	-	-	-	*O. intermedium*	UBA2471	-	-	-	GCA_002342125.1	Scaffold	4.07684	58	10	0
-	-	-	-	-	*Ochrobactrum* sp. UBA3110	UBA3110	-	-	-	GCA_002365515.1	Scaffold	4.57132	57.8	111	0
-	-	-	-	-	*Ochrobactrum* sp. UBA7634	UBA7634	-	-	-	GCA_002483905.1	Scaffold	4.54404	57.8	23	0

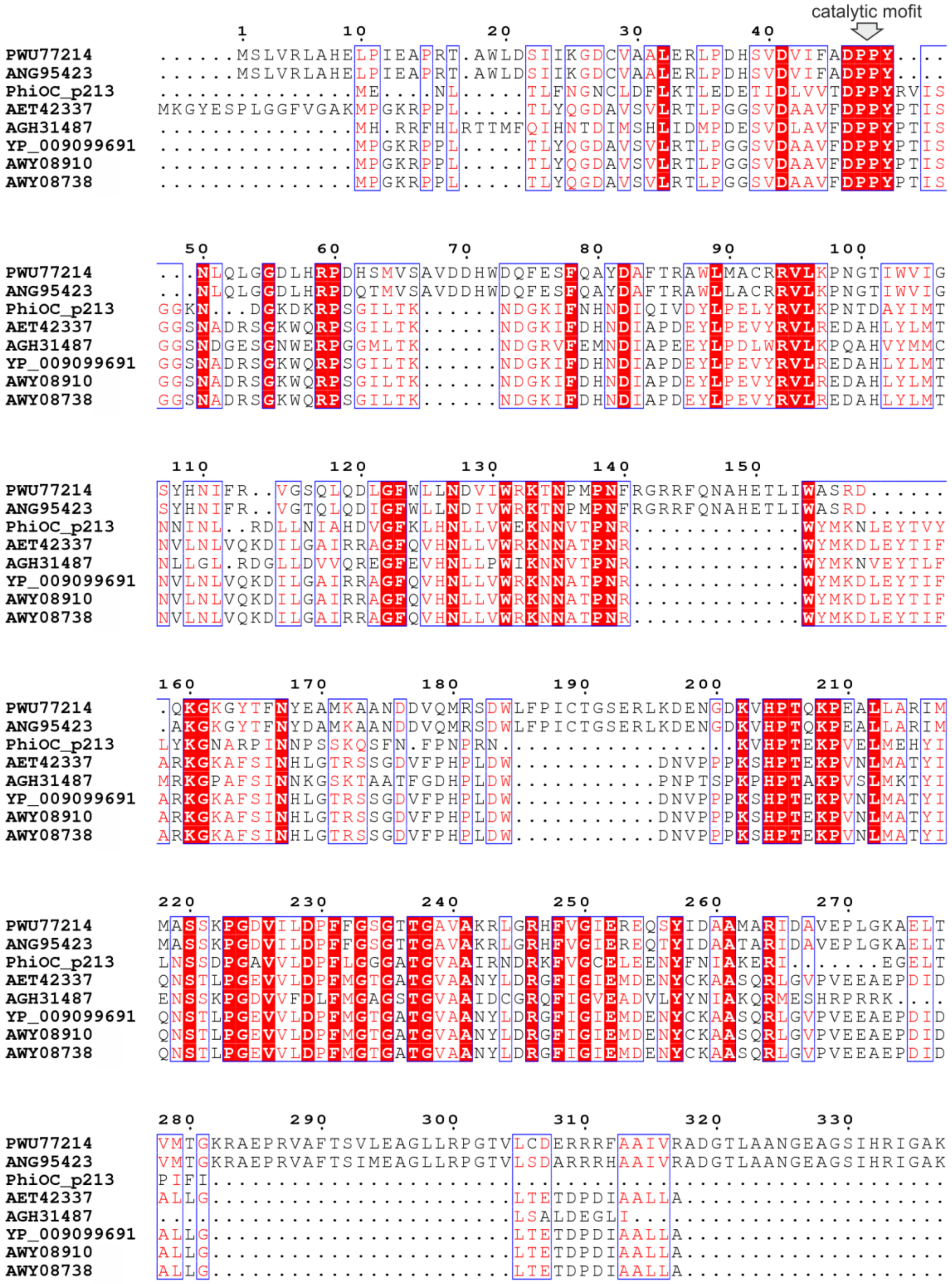

Figure S1. Multisequence alignment of CcrM and CcrM-like DNA methyltransferases. Those were acquired from the following organisms: *Ochrobactrum* sp. POC9 (GenBank acc. no. PWU77214), *O. pseudogrignonense* (GenBank acc. no. ANG95423), *Ochrobactrum* sp. POC9 vB_OspM_OC phage (PhiOC_p213), *Silicibacter* phage DSS3-P1 (GenBank acc. no. AET42337), *Loktanella* phage pCB2051-A (GenBank acc. no. AGH31487), *Ruegeria* phage DSS3-P1 (GenBank acc. no. YP_009099691), *Ruegeria* phage vB_RpoS-V18 (GenBank acc. no. AWY08910), *Ruegeria* phage vB_RpoS-V7 (GenBank acc. no. AWY08738). The alignment was prepared with Muscle [1] and visualized using ESPript v3.0 (http://espript.ibcp.fr) [2].

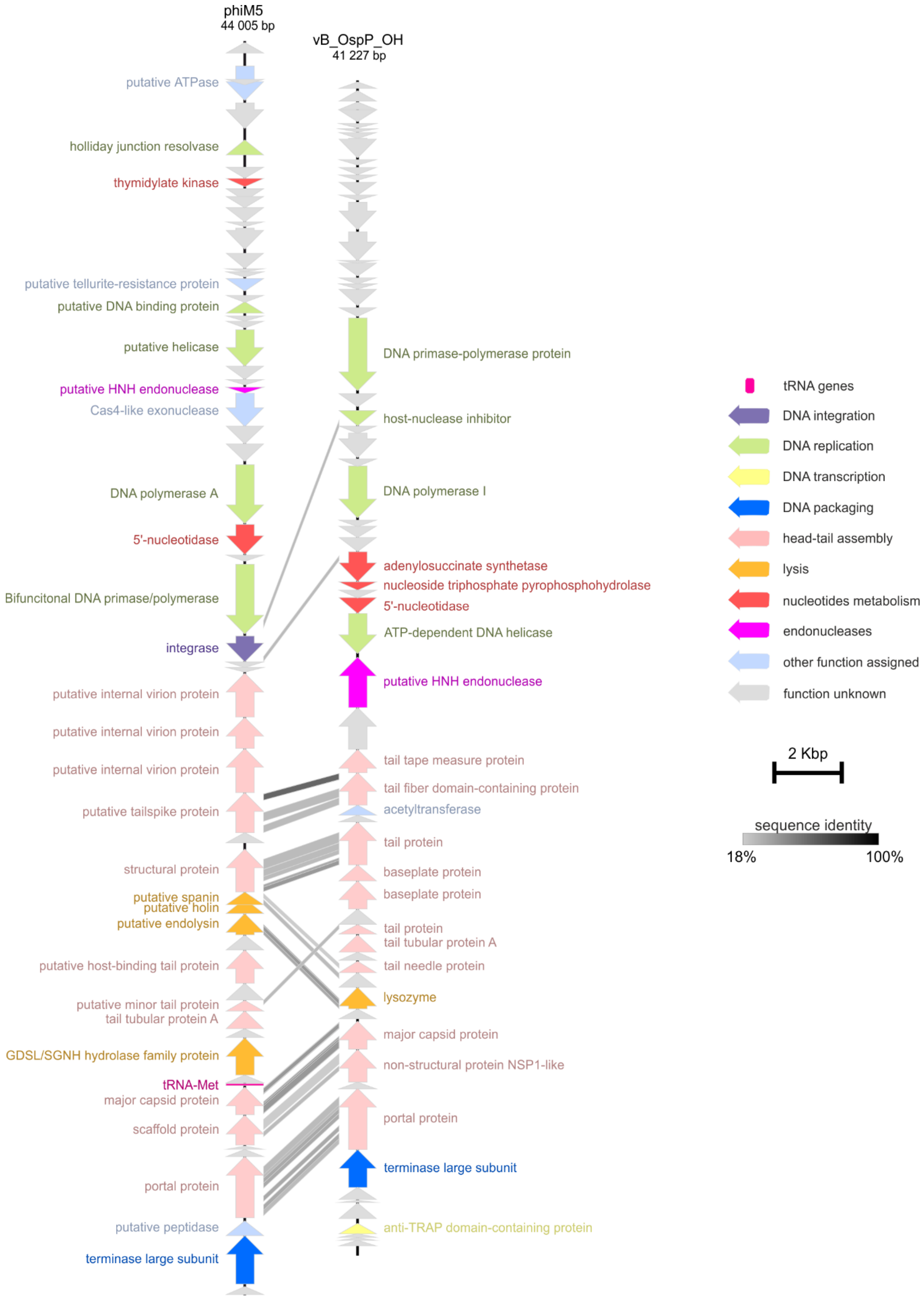

Figure S2. Genomic comparison of *Sinorhizobium* phage phiM5 and vB_OspP_OH. Proteins and tRNA gene encoded by both phages are represented as arrows and blocks, respectively, and colored according to the legend. The comparison was performed with EasyFig v2.2.2 [3] using tblastx with 1e-5 and minimum 50 amino acid alignment length as thresholds.

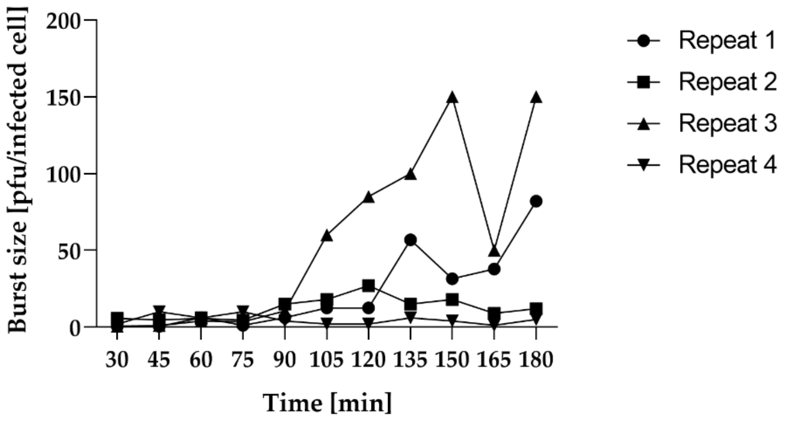

Figure S3. One-step growth curves of the vB_OspM_OC phage.

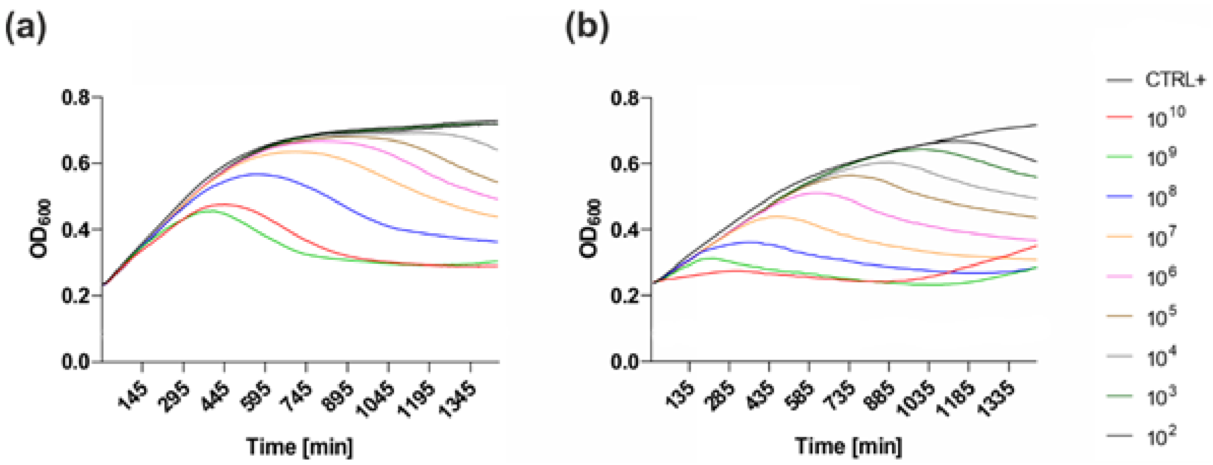

Figure S4. Bacterial culture collapse as the effect of phage infection. Panels **(a)** and **(b)** correspond to the infection with vB_OspM_OC and vB_OspP_OH, respectively. In legend, CTRL+ represents bacterial culture without the phage infection. The presented results are the average values from three experiments. The standard deviation between replicates were not presented to retain the transparency of plots presentation.
